# A ring of rotaxanes: studies of a large paramagnetic assembly in solution†

**DOI:** 10.1039/d3qi02165c

**Published:** 2023-11-02

**Authors:** Tom S. Bennett, Selina Nawaz, Selena J. Lockyer, Deepak Asthana, George F. S. Whitehead, Inigo J. Vitorica-Yrezabal, Grigore A. Timco, Neil A. Burton, Richard E. P. Winpenny, Eric J. L. McInnes

**Affiliations:** a Department of Chemistry, The University of Manchester Oxford Road Manchester M13 9PL UK eric.mcinnes@manchester.ac.uk richard.winpenny@manchester.ac.uk

## Abstract

Here we report the synthesis and structural characterization of four [7]rotaxanes formed by coordinating hybrid inorganic–organic [2]rotaxanes to a central {Ni_12_} core. X-ray single crystal diffraction demonstrate that [7]rotaxanes are formed, with a range of conformations in the crystal. Small angle X-ray scattering supported by molecular dynamic simulations demonstrates that the large molecules are stable in solution and also show that the conformers present in solution are not those found in the crystal. Pulsed EPR spectroscopy show that phase memory times for the {Cr_7_Ni} rings, which have been proposed as qubits, are reduced but not dramatically by the presence of the {Ni_12_} cage.

## Abstract 2

Here we report the synthesis and structural characterization of four [7]rotaxanes formed by coordinating hybrid inorganic–organic [2]rotaxanes to a central {Ni_12_} core.

## Graphical abstract

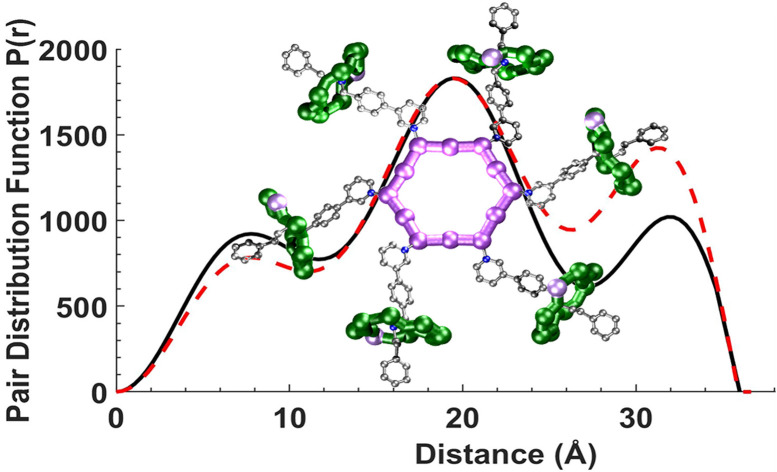

## Introduction

There is much interest in bringing together large molecules into still larger assemblies.^1–6^ Such assemblies have been studied as molecular containers for catalysis^7–12^ or as molecular machines or as complex molecular knots. Characterisation of such supramolecular assemblies invariably involves NMR spectroscopy as the solution method of choice. This becomes difficult, if not impossible, if the large assemblies contain components that are paramagnetic. Such paramagnetic supramolecules could, potentially, provide components for molecular machines and it has been proposed they could be used as multi-qubit arrays for quantum information. One unsolved question is how such paramagnetic assemblies could be characterised in solution. As the assemblies grow larger, techniques from materials science become applicable, such as dynamic light scattering. However, often there is little structural information found other than general information about the size of particles. This becomes a particularly acute problem if the large assembly can adopt multiple conformations. Alternatively, the assembly could be deposited on a surface and a technique such as AFM used to measure the size of assemblies.^6^ However, AFM is no longer looking at the assembly in solution.

Our particular interest in large assemblies involve heterometallic {Cr_7_Ni} rings which could be used as qubits for quantum information processing (QIP).^13,14^ Previous work includes routes to qubits linked by potential redox-switches,^15^ a report of a [13]rotaxane containing twelve {Cr_7_Ni} qubits^16^ and a demonstration that the qubits can be linked by Cu^II^ centres leading to a five-spin supramolecule that could be used to study decoherence in entangled states.^17^

One issue in our approach is that these large supramolecules could adopt multiple conformations in solution. Tiede *et al.* showed small angle X-ray scattering (SAXS) could be used for porphyrin based materials^18^ and recently we reported the solution structure of a rigid [13]rotaxane using a combination of SAXS and atomistic molecular dynamic simulations.^16^ We could also differentiate between structurally related [3] and [4]rotaxanes in solution.^19^ The question remains whether the technique is general and whether detailed structural information can be obtained from SAXS. Here we show that when combined with molecular dynamic simulations SAXS allows us to identify specific conformers of a [7]rotaxane in solution. This also identifies key structural requirements for such large supramolecules to be useful in QIP.

## Results and discussion

**Fig. 1:**
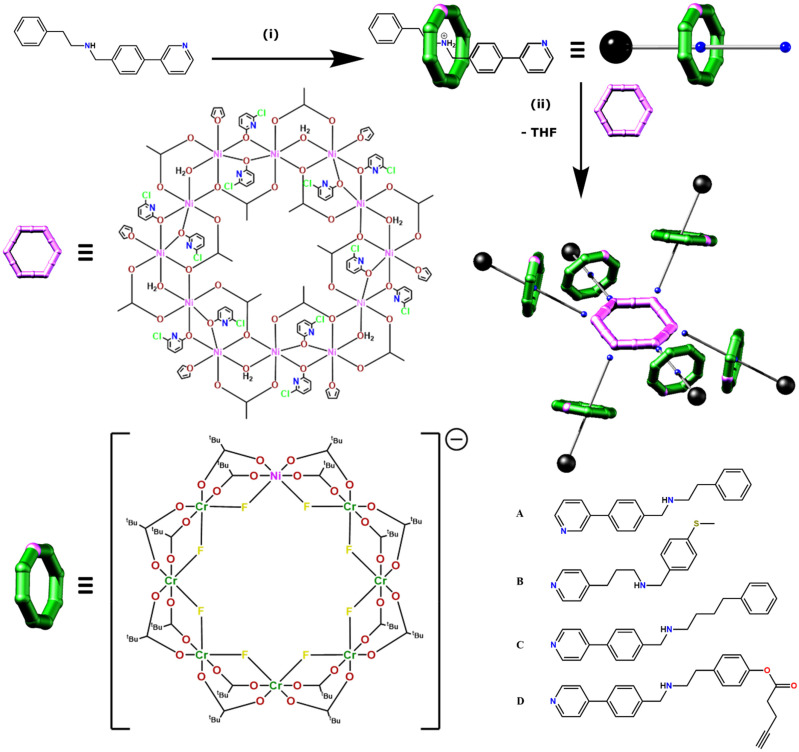
Synthesis of [7]rotaxanes. Generic synthetic route to a [7]rotaxane exemplified using 2-phenyl-*N*-(4-(pyridine-3-yl)benzyl) ethan-1-amine A as the thread in the parent [2]rotaxane to give 1A. Reaction conditions: (i) CrF_3_·4H_2_O, [Ni_2_(H_2_O)(O_2_C^*t*^Bu)_4_(HO_2_C^*t*^Bu)_4_], ^*t*^BuCO_2_H (140–160 °C, 24 h). (ii) [2(THF)_6_], toluene (60 °C, 10 min) alongside the four threads used in the synthesis of [7]rotaxanes (A–D).

The family of [7]rotaxanes is based on hybrid inorganic–organic [2]rotaxanes, where the ring of the rotaxane is an inorganic heterometallic octagon, [Cr_7_NiF_8_(O_2_C^*t*^Bu)_16_]^−^, and the thread is an organic molecule with a central ammonium cation about which the inorganic ring is grown, and which is terminated at one end by a pyridine group (Fig. 1). The threads used in this work are given in Fig. 1b. These [2]rotaxanes can then act as ligands for further metal cages, allowing us to develop families of compounds straightforwardly. For this paper we label the [2]rotaxanes as 1X, where X = the identity of the thread. The general formulae are then [X@Cr_7_NiF_8_(O_2_C^*t*^Bu)_16_]. At the end of thread A the binding group is a 3-pyridyl while in threads B, C and D the binding group is a 4-pyridyl (Fig. 1). B also differs in having a fully saturated chain between the pyridyl and the ammonium centre about which the {Cr_7_Ni} ring grows. These [2]rotaxanes are stable in solution unless strong base is present.

The [2]rotaxanes were coordinated to a dodecametallic nickel(ii) ring [Ni_12_(chp)_12_(O_2_CMe)_12_(H_2_O)_6_(THF)_6_] [2(THF)_6_] (Hchp = 6-chloro-2-hydroxypyridine) (Fig. 1) which has Ni^II^ sites at the corners and mid-points of the edges of a hexagon.^20,21^ [2(THF)_6_] is insoluble in any solvent but dissolves in toluene at 60 °C in the presence of six equivalents of 1X to give a series of [7]rotaxanes, [2(1X)_6_].

**Fig. 2:**
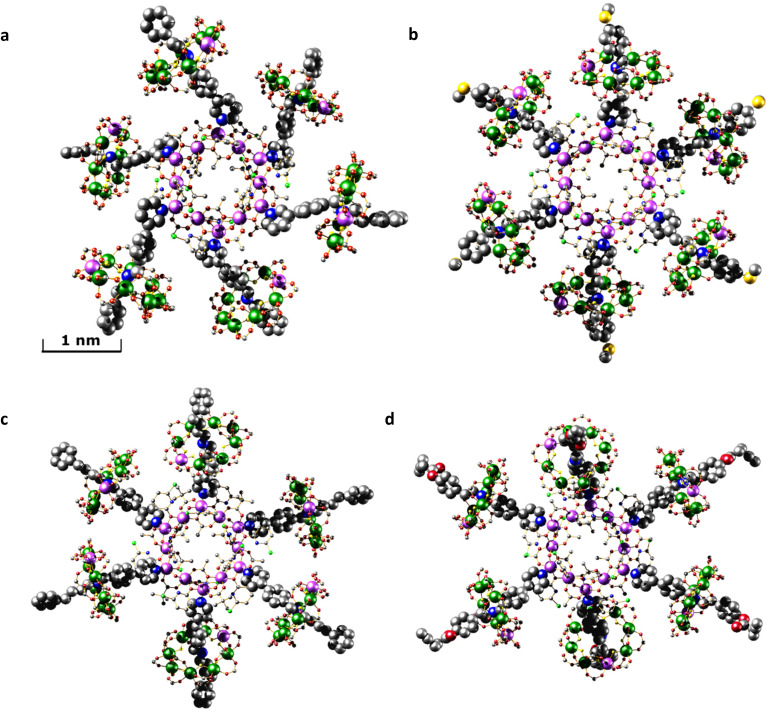
Single crystal X-ray structures. (a) [2(1A)_6_]. (b) [2(1B)_6_]. (c) [2(1C)_6_]. (d) [2(1D)_6_]. Images are displayed perpendicular to the plane of the {Ni_12_} ring. Colour scheme: Cr, dark green; Ni, lilac; O, red; N, blue; C, grey; F, yellow; S, gold; Cl, bright green. H-atoms and ^*t*^Bu groups of pivalates have been omitted for clarity. The Ni sites in the {Cr_7_Ni} rings are disordered over multiple positions.

Crystallography shows that in each compound the six [2]rotaxanes bind to the nickel(ii) sites at the corners of the hexagon (Fig. 2). Collecting high resolution data on crystallographic samples of this nature is difficult on account of the size of the molecules and inherent disorder within the structure. Despite the use of synchrotron radiation, higher resolution data could not be collected. The diffracting strength of these samples drops off rapidly at higher angles, akin to protein structures of similar size. Care has been taken in the creation of the structural models to ensure that no egregious assumptions have been made and to limit the use of constraints in refining the model. The assumptions made are that similar moieties will have similar metrical parameters, and so similar organic moieties have been refined to have similar 1,2- and 1,3-bond distances. In limited cases, fixed distance restraints or idealised constraints for phenyl rings, a common practice, have been employed for poorly defined groups at the end of the threads. For the majority of refined sites, atomistic resolution is achieved, and while there is a lack of definition on the electron density map toward the periphery of the molecules (Fig. S1–S4†) we feel these models, any assumptions made, and the modelling techniques used are justified for the data obtained.

Within the central {Ni_12_} ring the Ni⋯Ni edges are alternately bridged in two distinct ways; one edge is bridged by two μ_2_-O from chp^−^ and a bridging acetate that lies within the ring. The second edge is bridged by a μ_2_-water and a μ_2_-O from acetate and a bridging acetate that lies outside the ring.

The THF attached to 2 is displaced by 1X in each case and the structures of 1X are unchanged on binding. For molecule [2(1C)_6_] the structure of the central {Ni_12_} ring subtly changes during the coordination of [2]rotaxane 1C. Four of the six external acetate bridging ligands are displaced with pivalate ligands. Ligand exchange from the surrounding {Cr_7_Ni} rings is the likely source of the pivalate ligands on the central {Ni_12_} ring. The exact location of the exchanged pivalate ligands are localised and are two easily accessible sites that are not involved in any μ_2_-O bridging. For structure [2(1A)_6_] a chp ligand has been exchanged with an acetate ligand, although the extent of this substitution disorder is difficult to determine crystallographically; one site could be satisfactorily modelled as disordered, but all sites show some evidence of substitution.

The conformations of the [7]rotaxanes in the crystal vary: we give three ranges in Table 1 and more detail in Table S2.† The {Cr_7_Ni} ring centroid to the Ni^II^ site of 2 to which it is bound *via* the thread is fairly consistent within each [7]rotaxane, and is dependent on chain length of the thread, and the substitution pattern at the pyridine. The nearest neighbour centroid⋯centroid cover a wider range, and are noticeably more varied in [2(1C)_6_] and [2(1D)_6_] than in [2(1A)_6_] or [2(1B)_6_]. The [7]rotaxanes are not isotropic, and their diameter also has a range: [2(1A)_6_] or [2(1B)_6_] are the smallest and have the least variation; [2(1D)_6_] is 10 Å bigger and has a greater variation depending on where the diameter is measured. Similar variations are also seen in the angle that the thread itself makes with respect to the mean plane of 2 (Table S2†) and in the angle between the planes of the {Cr_7_Ni} rings and the {Ni_12_} ring. Here again [2(1A)_6_] or [2(1B)_6_] are similar, with two angles around 64° and four angles between 70 and 80°; in [2(1C)_6_] and [2(1D)_6_] one angle is around 32° and four between 80 and 90°. These are subtle changes which are presumably due to crystal packing as there seems no obvious steric explanation.

**Table 1:** Distance ranges (Å) in the four [7]rotaxanes and angles between planes of rings (°) in crystal structures (CS) and optimised conformer (OC) from molecular dynamic simulations. Estimated standard deviations on bonds <0.01 Å and on angles <0.1°

	[2(1A)_6_]		[2(1B)_6_]		[2(1C)_6_]		[2(1D)_6_]	
	CS	OC	CS	OC	CS	OC	CS	OC
{Cr_7_Ni} centroid–Ni{Ni_12_}	10.57 ± 0.19	10.24 ± 0.02	9.53 ± 0.07	9.15 ± 0.02	11.30 ± 0.06	10.45 ± 0.01	11.15 ± 0.04	10.23 ± 0.01
{Cr_7_Ni} centroid–{Cr_7_Ni} centroid	32.21 ± 0.68	25.89 ± 0.37	30.49 ± 0.21	28.24 ± 0.17	33.65 ± 1.27	31.91 ± 1.43	33.32 ± 1.46	30.72 ± 0.64
Diameter of [7]rotaxane	44.03 ± 0.51	33.88 ± 0.43	45.49 ± 0.33	35.89 ± 1.18	50.90 ± 1.44	48.43 ± 0.24	56.24 ± 1.55	58.66 ± 0.21
Angle between planes of 1X and 2	62.4	57.3	66.1	17.7	35.7	34.0	29.4	47.5
	78.4	59.3	70.5	18.5	79.6	36.2	80.9	49.5
	79.7	58.2	75.0	88.7	89.2	36.5	87.0	50.0
		86.7		88.8		67.8		55.3
		88.5		89.3		70.1		57.3
		89.3				70.4		57.7

Several other threads were studied but the resolution of the diffraction data obtained was too low to report the structures.

### SAXS studies

**Fig. 3:**
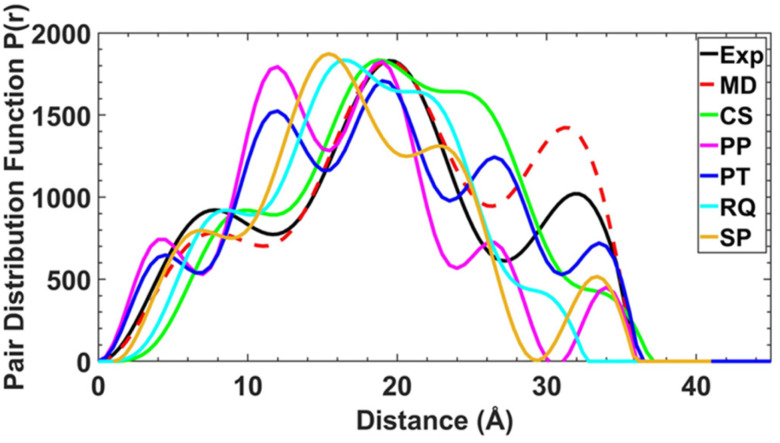
SAXS data shown as pair–pair distributions for select conformers of [2(1A)_6_] (pink, blue, cyan, orange – see ESI† for details of individual conformers) alongside the crystal structure (CS, bright green), experimental (black) and final MD result (red).

To determine whether the structures are maintained in solution, and to examine the conformational flexibility in the [2(1X)_6_] family, we measured SAXS data on all four [7]rotaxanes (Fig. 3). Each of the experimental SAXS data show three main peaks which vary in intensity depending on the structure. [2(1A)_6_] shows major maxima at around 8, 20 and 33 Å, with the pairwise distribution function (PDF) falling to zero around 36 Å. The longest distance is more prominent in this molecule than for [2(1B)_6_], [2(1C)_6_] and [2(1D)_6_].

An attempt was made to use the molecular geometries adopted in the crystal structure as a starting point to calculate theoretical SAXS data for comparison. However, the experimental SAXS data for each of the [7]rotaxanes are not consistent with the conformers adopted in the crystal (Fig. S12–S15†).

To attempt a better fit of the experimental SAXS data, 100 unique initial static conformations across the four molecules were assessed for viability (see ESI† for discussion of structural degrees of freedom). Density functional theory (DFT) was used to optimise the thread, {Cr_7_Ni} ring and 2 independently. The magnetic exchange interactions in 1X and 2 were set as anti-ferromagnetic and ferromagnetic respectively, based on previous studies of the magnetic behaviour of the components.^13,20^ In order to aid selection of feasible conformers, a primitive distance-based scoring function was used to assess the number and severity of the bad-contacts (short non-bond interactions due to close proximity or overlap of functional groups) in each model.

**Fig. 4:**
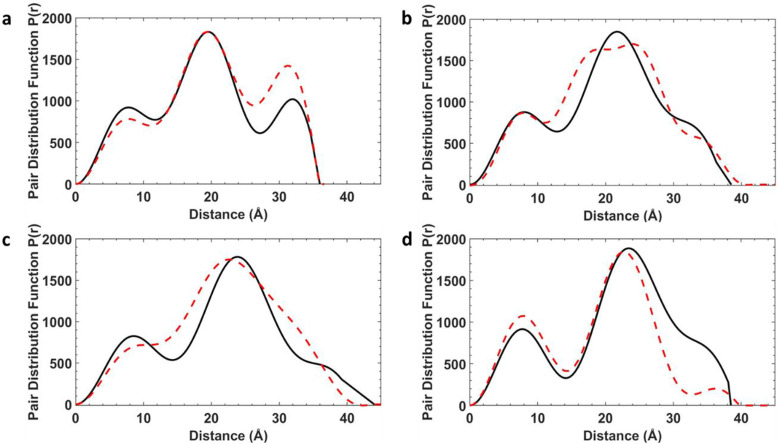
SAXS data for [7]rotaxanes. (a) [2(1A)_6_]. (b) [2(1B)_6_]. (c) [2(1C)_6_]. (d) [2(1D)_6_], experimental SAXS data (black) and MDS (red) as pairwise distribution functions. All data and simulations are in toluene at 293 K.

For these static conformations the SAXS spectrum and its corresponding electron pair–pair distribution function *P*(*r*), were then calculated for each of the four [7]rotaxanes (Fig. 3 for [2(1A)_6_] and Fig. S12–S15†). Each of the conformations may be found in solution, but due to steric hinderance, have relatively little freedom to interconvert between conformers without dissociating so to do. The single conformer which showed the best agreement between these calculated SAXS data and the experimental SAXS data was then chosen as the basis for a molecular dynamics simulation (MDS). Fig. 4 shows a comparison of the experimental and theoretical SAXS data for all four of the [7]rotaxanes after the MDS step, and in each case the experimental SAXS and the SAXS calculated for MD averaged structure match well.

Calculations on the low energy static conformers without the MDS are less consistent with the experimental observations as the electron distribution is significantly higher at certain distances. These extra peaks are averaged in the MD and experimental SAXS data.

**Fig. 5:**
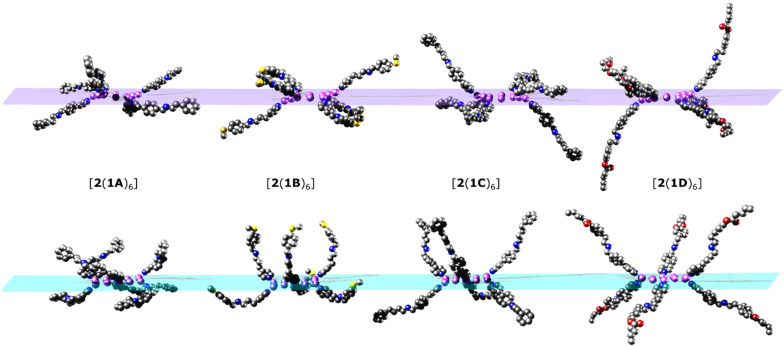
Simplified structures showing just the coordinated threads, viewed with the {Ni_12_} ring perpendicular to the plane of the paper. Top: conformers from the crystal structures. Bottom: optimised conformers used for the MD simulations. {Cr_7_Ni} rings and other ligands attached to the {Ni_12_} ring have been omitted for clarity. Colour scheme as in Fig. 2.

As there is little freedom to interconvert between each of the low energy conformations without dissociation, each conformer from which MDS were performed has been compared to the crystal structures (Fig. 5). Significant curvature of threads within the crystal is less likely than in solution due to favourable intermolecular packing interactions between [7]rotaxanes. For this reason, each calculated conformer contains shorter {Cr_7_Ni}–{Cr_7_Ni} distances (Table 1) than in the crystal structure. The diameter of each molecule is also smaller in the calculated conformers for [2(1A)_6_], [2(1B)_6_], [2(1C)_6_] but slightly larger in [2(1D)_6_].

Conformational flexibility of the thread leads to large differences in the angles between mean planes of 1X and 2 when comparing optimised structures with those observed in the crystal (Tables 1 and S2†). The angle of pyridyl coordination to the {Ni_12_} plane is consistent across the optimised structures and differs very little from those measured in the crystal structure (Table S2†).

Comparison of the optimised and crystal conformers highlights the large differences in the size and shape of each molecule available in solution. While each [7]rotaxane shows a change between crystal and solution phase, there is perhaps least change in [2(1C)_6_] (Table 1), with a similar diameter and angles between the planes of the rings. Multiple conformations of each molecule are likely to exist in solution, contributing to a wide range of metal–metal distances. Agreement between the theoretical and experimental SAXS data suggest that these large [7]rotaxane structures are stable in solution, but consideration of the dynamic behaviour of such molecules is important in the interpretation of SAXS data.

### EPR studies

We have proposed that such multiple ring assemblies could be used in quantum information processing.^13–17,19,22^ One key question in such complex molecules is the coherence times of the proposed qubits; the presence of other paramagnetic units may offer a mechanism for decoherence beyond nuclear hyperfine interactions.^23–27^ As [2(THF)_6_] is a single molecule magnet^20^ with an *S* = 12 ground state and a very low thermal energy barrier for magnetic relaxation, the [7]rotaxanes allow us to study this in an extreme case.

**Fig. 6:**
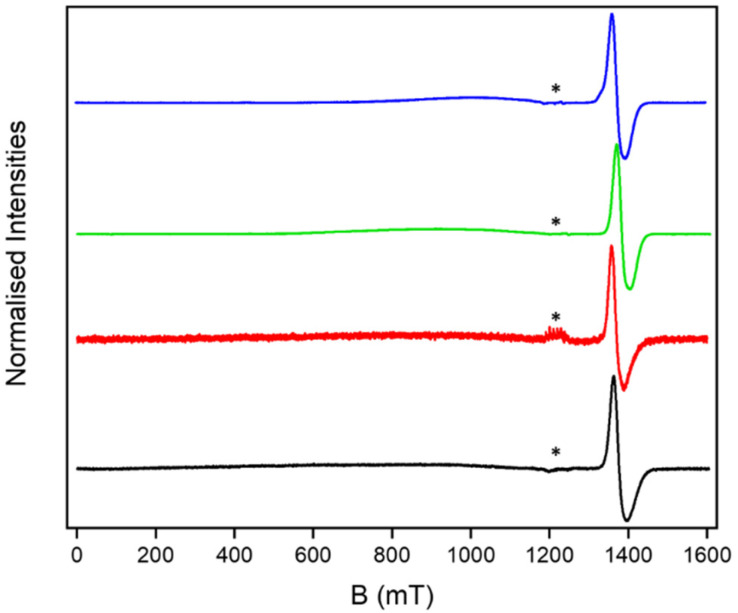
Solution cw Q-band EPR for [2(1A)_6_] (black), [2(1B)_6_] (red), [2(1C)_6_] (green) and [2(1D)_6_] (blue) at 5 K. * indicates a Mn^II^ impurity in the EPR tube.

The continuous wave (cw) EPR spectra of the [2(1X)_6_] [7]rotaxanes are all similar (Fig. 6). A resonance is seen at around *g* = 1.8, which is typical for a {Cr_7_Ni} ring. A very broad, barely noticeable resonance is also seen centred at 1000 mT and has a line-width of at least 400 mT. We measured the EPR of [2(THF)_6_] as a powder and found the same very broad resonance centred at 1000 mT consistent with the very high density of states in this *S* = 12 unit (Fig. S17†).

The solution studies performed by SAXS indicate that the [7]rotaxanes are stable in solution, and therefore this family of molecules give us the opportunity to study how the presence of an *S* = 12 SMM influences the relaxation of the qubit. Therefore we studied the spin–lattice (*T*_1_) and phase memory (*T*_m_) relaxation times for the {Cr_7_Ni} rings in the [2(1X)_6_] rotaxanes (see ESI† for methods used). The results measured at the maximum of the absorption in the field-swept echo-detected spectra are given in Table 2.

**Table 2:** Q-band relaxation times for the {Cr_7_Ni} components of [2(1X)_6_] measured at 3 K in 0.1 mM solution in toluene

Compound	*T*_m_/ns	*T*_1_/μs
[2(1A)_6_]	500 (0.5)	142 (1.0)
[2(1B)_6_]	446 (0.5)	116 (1.0)
[2(1C)_6_]	457 (0.5)	106 (1.0)
[2(1D)_6_]	414 (0.5)	123 (0.5)
3	713 (0.42)	62 (0.38)
4	826 (0.56)	108 (0.75)
5	425 (2)	27 (0.3)

To understand the *T*_m_ and *T*_1_ times it is useful to compare them with measurements on related rotaxanes. We have reported [4]- and [3]-rotaxanes [CrNi_2_F(1E)_3_] 3 and [CrNi_2_F(1E)_2_(THF)] 4 (where E is a slightly shorter thread py-CH_2_CH_2_NHCH_2_C_6_H_4_SCH_3_) involving a {CrNi_2_} triangle with an *S* = 1/2 ground state as the central link,^19^ and {[Pd_6_[HF{Cr_7_NiF_8_(O_2_C^*t*^Bu)_16_}]_12_] [[Cr_7_NiF_8_(O_2_C^*t*^Bu)_16_]_6_(BF_4_)_6_]} 5 (where F = PhCH_2_CH_2_NHCH_2_–C_6_H_4_–C_6_H_4_–OH), which has eighteen rings around a *diamagnetic* core.^16^ The relaxation times for the {Cr_7_Ni} in 3, 4 and 5 are given in Table 2 as a comparison for the [2(1X)_6_] rotaxanes. The *T*_m_ value in the [7]rotaxanes, in the presence of the *S* = 12 {Ni_12_} ring, is shorter than in 3 and 4, with an *S* = 1/2 {CrNi_2_} triangle.^19^ However, it is similar to that in 5.^16^ Hence, the shorter *T*_m_ of the [7]rotaxanes may be simply due to the higher local spin concentration (*via* instantaneous diffusion) due to the greater number of {Cr_7_Ni} rings in these supramolecules.

It is also noticeable that *T*_1_ for 4, which is conformationally flexible, is close to that found for the flexible [7]rotaxanes we have studied here, while the rigid [4]rotaxane 3 has a shorter *T*_1_.^19^5 also has a shorter *T*_1_, and this structure is rigid in solution as judged by an excellent agreement between X-ray diffraction and SAXS data.^16^ This very small data set suggests *T*_1_ is reporting on rigidity in these large supramolecules.

The similar *T*_m_, and shorter *T*_1_, for 5*cf*. the [7]rotaxanes imply that the {Ni_12_} core of the latter is not having a significant influence on the relaxation properties of {Cr_7_Ni}. The interaction between the {Ni_12_} and {Cr_7_Ni} rings must be in the weak regime with regard to the difference in resonance frequencies (also consistent with cw spectra). The similar *T*_m_ could mean that *T*_1_ of {Cr_7_Ni} (*T*_1_ ≈ 10^2^ μs; 1/*T*_1_ ≈ 0.01 MHz) is faster than that of {Ni_12_}, hence is unaffected by the latter. If {Ni_12_} is faster relaxing, then its *T*_1_ must still be slower than the interaction frequency such that it has minimal effect on *T*_m_ of the slower relaxing {Cr_7_Ni}.^28^ We can very crudely estimate an interaction of 50 MHz, based on a Ni⋯{Cr_7_Ni} centroid distance of 10 Å in the point dipole approximation. This implies that 1/*T*_1_ of {Ni_12_} would be in the MHz regime (*T*_1_ in the μs regime). 1/*T*_1_ relaxation rates for {Ni_12_} have only been measured in the solid state (by ac susceptibility) and at 0.5 K and below.^20^

SAXS is measured on more concentrated solutions than the dilute solutions used for pulsed EPR spectroscopy. Therefore there remains a possibility that the echo we are measuring is due to a small component of 1X that is dissociating. The change in relaxation times between the [2(1X)_6_] family and 3 and 4, and the similarity to those of 5, supports the proposition that dissociation is not occuring.

## Conclusions

If complex supramolecules were to be used in quantum technology two key questions are whether such supramolecules could be reproducibly deposited or crystallised from solution without changes in conformation; our SAXS studies here suggest that for this family of [7]rotaxanes this would be a problem. The single coordinate bond between a Ni^II^ ion in 2 and an N-donor from 1X allows flexibility in solution. A second key question is whether proximity to paramagnetic units may cause decoherence in the qubits. The evidence we have is that presence of the *S* = 12 {Ni_12_} ring shortens phase memory times for 1X but not catastrophically.

Therefore, if large assemblies are to be used for QIP they need to be more rigid and involve stronger bonds between the components. In a [13]rotaxane,^16^ where there were two Pd^II^–N bonds attaching the {Cr_7_Ni} rings to the core we found no change between solution conformation and the crystal structure, and clearly that is a better design going forward. In future work we will focus on rigid structures. More inert bonds between the qubits and the central core of the supramolecular will also be advantageous.

## Author contributions

T. S. B. and D. A. performed the synthesis advised by G. A. T. Crystallographic studies were carried out by T. S. B., G. F. S. W. and I. J. V.-Y. The MD and DFT calculations were performed by S. N. and N. D. B. EPR spectra were measured and analysed by S. J. L. The project was designed by E. J. L. M. and R. E. P. W. The manuscript was written by T. S. B., E. J. L. M. and R. E. P. W. with input from all authors.

## Conflicts of interest

The authors declare no competing financial interest.

## Supplementary Material

QI-010-D3QI02165C-s001

QI-010-D3QI02165C-s002
