# Supplementary material for: A ring of rotaxanes: studies of a large paramagnetic assembly in solution
Source: Inorg Chem Front. 2023 Nov 2;10(23):6945–52. doi: 10.1039/d3qi02165c (PMC10660382; doi:10.1039/d3qi02165c)
Supplement: QI-010-D3QI02165C-s001 [file QI-010-D3QI02165C-s001.pdf]

Electronic Supplementary Material (ESI) for Inorganic Chemistry Frontiers.
This journal is © the Partner Organisations 2023

Supplementary Information

A Ring of Rotaxanes: Studies of a Large Assembly in Solution

Tom S. Bennett, Selina Nawaz, Selena J. Lockyer, Deepak Asthana, George F.S. Whitehead, Inigo J. Vitorica-Yrezabal, Grigore A. Timco, Neil A. Burton, Richard E. P. Winpenny* and Eric J. L. McInnes*

Contents

SYNTHESIS 2

CRYSTALLOGRAPHY 10

SMALL ANGLE X-RAY SCATTERING 19

ATOMISTIC MOLECULAR DYNAMICS SIMULATIONS 19

EPR SPECTROSCOPY 33

REFERENCES 37

Synthesis

[$Ni_{12}(chp)_{12}(O_2CMe)_{12}(H_2O)_6(THF)_6$] (2): Prepared using the method given in H. Andres, *et al*, Studies of a nickel-based single molecule magnet. *Chem. Eur. J.* **8**, 4867 – 4876 (2002).

2-(3-(pyridin-4-yl)propyl)isoindoline-1,3-dione and 3-(pyridin-4-yl)propan-1-amine. Prepared using the method given in J. Disch *et al*, Discovery of thieno[3,2-d]pyrimidine-6-carboxamides as potent inhibitors of SIRT1, SIRT2, and SIRT3. J. Med. Chem. **56**, 3666 – 3679 (2013).

3-(4-bromophenyl)pyridine Prepared using the method given in T. Kim *et al*, Octahedral [Pd_6L_8]$^{12+}$ Metallosupramolecular Cages: Synthesis, Structures and Guest-Encapsulation Studies. Chem. Eur. J. **23**, 15089 – 15097 (2017).

4-phenyl-N-(4-(pyridin-4-yl)benzyl)butan-1-amine (C). Prepared using the method given in S. J. Lockyer, *et al*, Close Encounters of the Weak Kind: Investigations of Electron− Electron Interactions between Dissimilar Spins in Hybrid Rotaxanes. *J. Am. Chem. Soc.*, 2019, **141**, 14633–14642.

[$C_{22}H_{25}N_2$][$Cr_7NiF_8(O_2C^tBu)_{16}$] (1C): Prepared using the method given in S. J. Lockyer, *et al*, Close Encounters of the Weak Kind: Investigations of Electron− Electron Interactions between Dissimilar Spins in Hybrid Rotaxanes. *J. Am. Chem. Soc.*, 2019, **141**, 14633–14642.

2-phenyl-N-(4-(pyridin-3-yl)benzyl)ethan-1-amine (A)

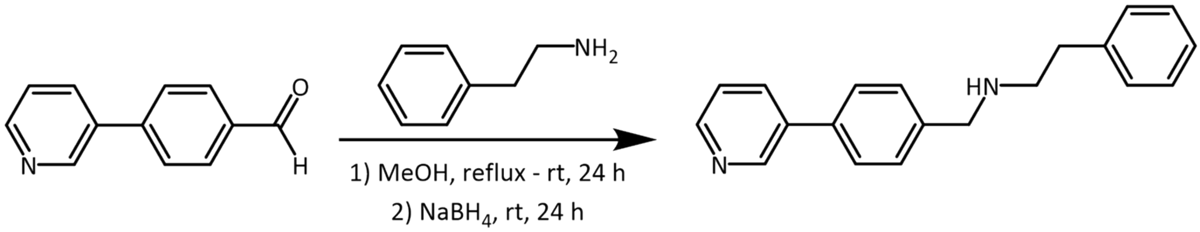

4-(Pyridin-3-yl)benzaldehyde (3.49 g, 19.07 mmol, 1.1 eq) was dissolved in 200 mL of methanol and purged under a N_2 atmosphere for 1 h. Phenylethylamine (2.18 mL, 17.34 mmol, 1 eq) was added to the solution with stirring and refluxed for 5 h under an N_2 atmosphere before cooling to room temperature and left to stir overnight. $NaBH_4$ (3.28 g, 86.7 mmol, 5 eq) was added slowly to the solution before stirring overnight to reduce the imine. The reaction was quenched with 100 mL of water and solvent removed under a reduced pressure. The resultant solid was redissolved in 200 mL of $CHCl_3$ and washed with water (3 x 70mL) and dried over anhydrous magnesium sulphate and filtered. The solvent was removed under a reduced pressure yielding a white solid (4.11 g, 82 %). ^1H δ ppm (400 MHz; $CDCl_3$): 2.82 (t, 2H), 2.88 (t, 2H), 3.81 (s, 2H), 7.09 – 7.53 (m, 10H), 8.50 (dd, 1H), 8.75 (d, 1H).

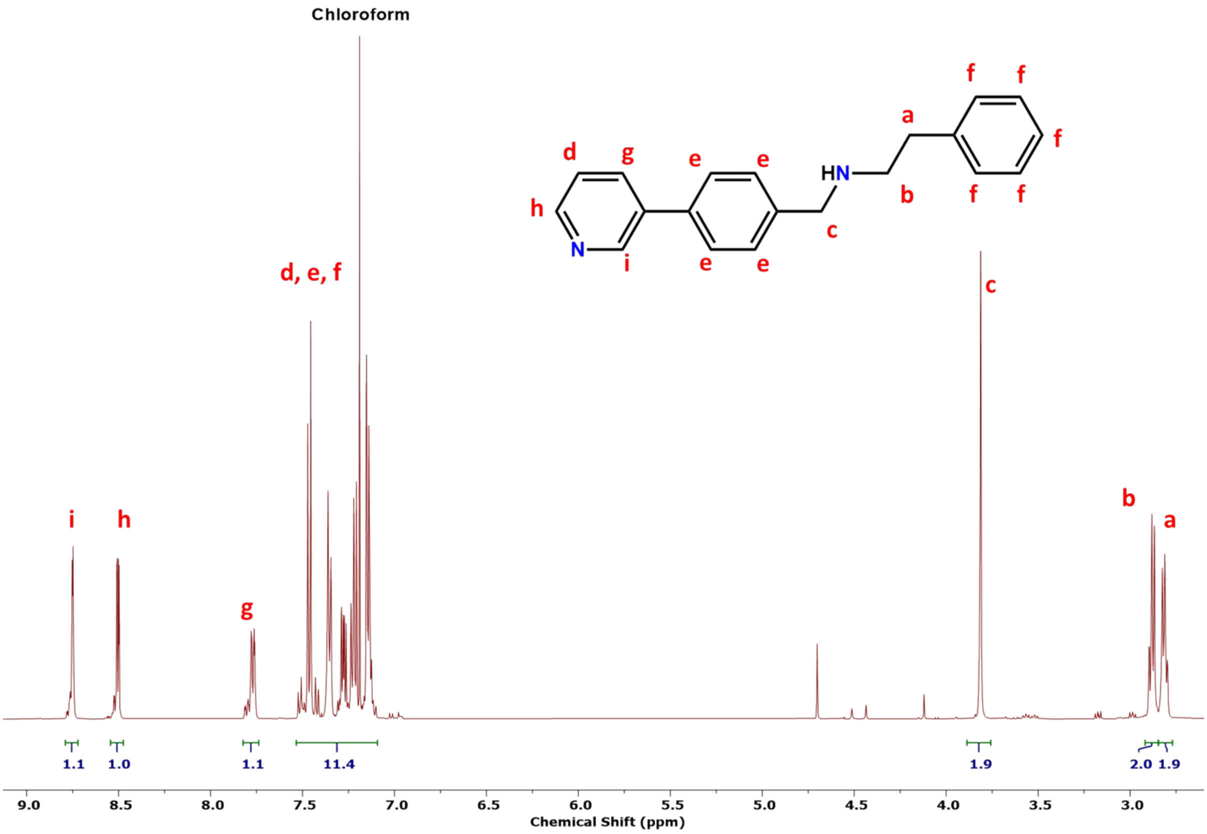

N-(4-(methylthio)benzyl)-3-(pyridin-4-yl)propan-1-amine (B)

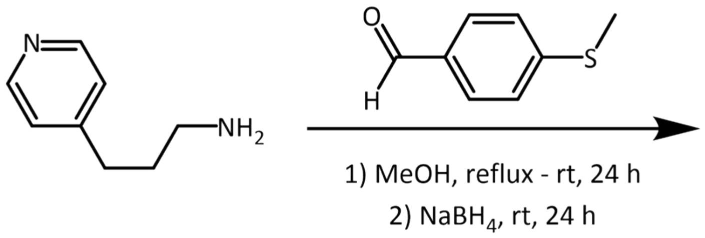

3-(pyridin-4-yl)propan-1-amine (1.00 g, 7.34 mmol, 1 eq) was dissolved in 80 mL of methanol and purged under a N_2 atmosphere for 1 h. 4-(Methylthio)benzaldehyde (1.08 mL, 8.08 mmol, 1.1 eq) was added to the solution with stirring and refluxed for 5 h under an N_2 atmosphere before cooling to room temperature and left to stir overnight. $NaBH_4$ (1.39 g, 36.71 mmol, 5 eq) was added slowly to the solution before stirring overnight to reduce the imine. The reaction was quenched with 50 mL of water and solvent removed under a reduced pressure. The resultant solid was redissolved in 150 mL of $CHCl_3$ and washed with water (3 x 50mL) and dried over anhydrous magnesium sulphate and filtered. The solvent was removed under a reduced pressure yielding a white solid (1.79 g, 90 %). ^1H δ ppm (400 MHz; $CDCl_3$): 1.91 (quint, 2H), 2.49 (s, 3H), 2.69 (m, 4H), 3.79 (s, 2H), 7.09 – 7.13 (m, 2H), 7.22 – 7.27 (m, 2H), 7.29 – 7.35 (m, 2H), 8.50 (dd, 2H).

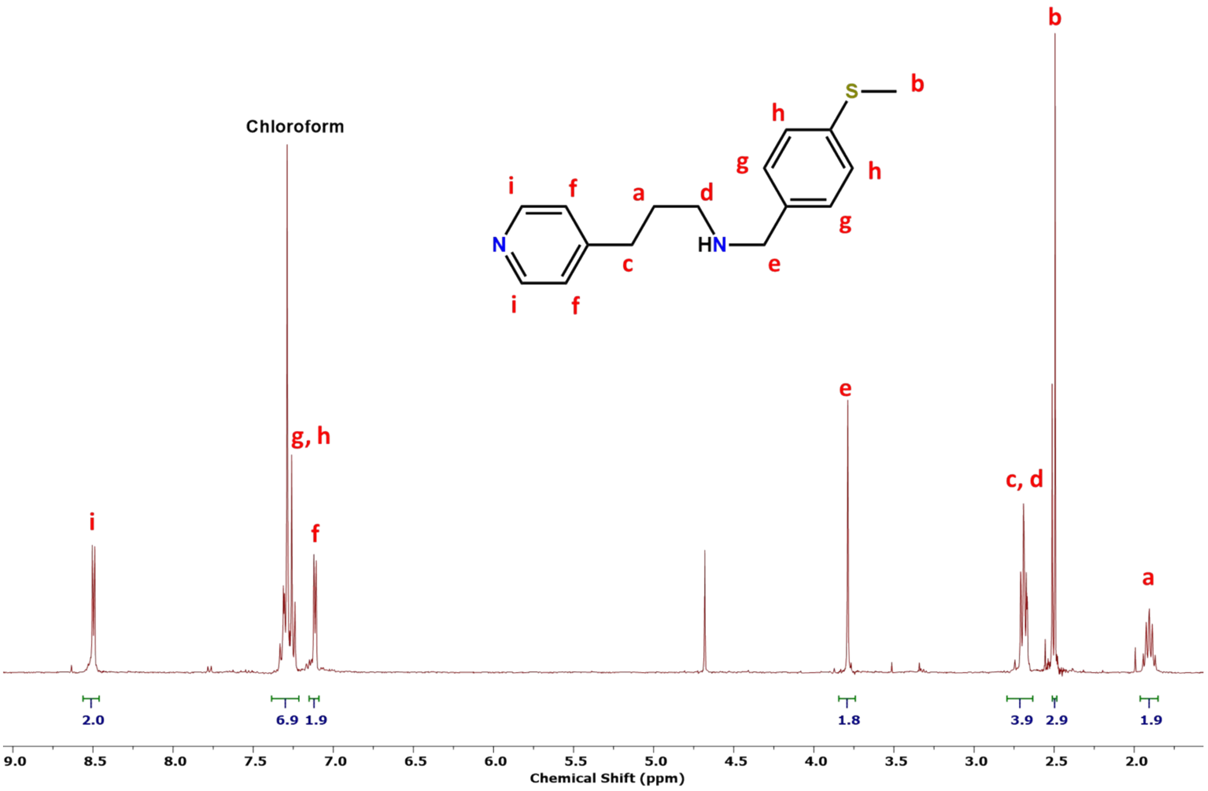

4-(2-((4-(pyridin-4-yl)benzyl)amino)ethyl)phenol (E)

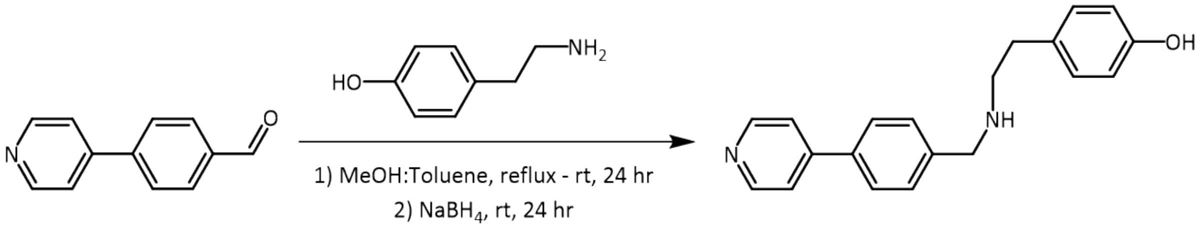

4-(pyridin-4-yl)benzaldehyde (1.525 g, 8.324 mmol, 1 eq) and 4-(2-aminoethyl)phenol (1.25 g, 9.12 mmol, 1eq) were dissolved in 50 ml MeOH and 30 ml toluene with stirring and refluxed for 4h under a N_2 atmosphere. The reaction was allowed to cool to room temperature before slow addition of $NaBH_4$ (1.19 g, 31.5 mmol, 3.8 eq) and allowed to stir overnight in order to reduce the imine. The reaction was quenched with 15 mL of water and solvent removed under a reduced pressure. The resultant solid was filtered and washed with 50 mL of chloroform to yield a white solid (1.50 g, 59 %). ^{1}H δ ppm (400 MHz; DMSO-d_6): 2.64 (m, 4H), 3.77 (d, 2H), 6.65 (d, 2H), 6.98 (d, 2H), 7.46 (d, 2H), 7.73 (m, 4H), 8.62 (d, 2H).

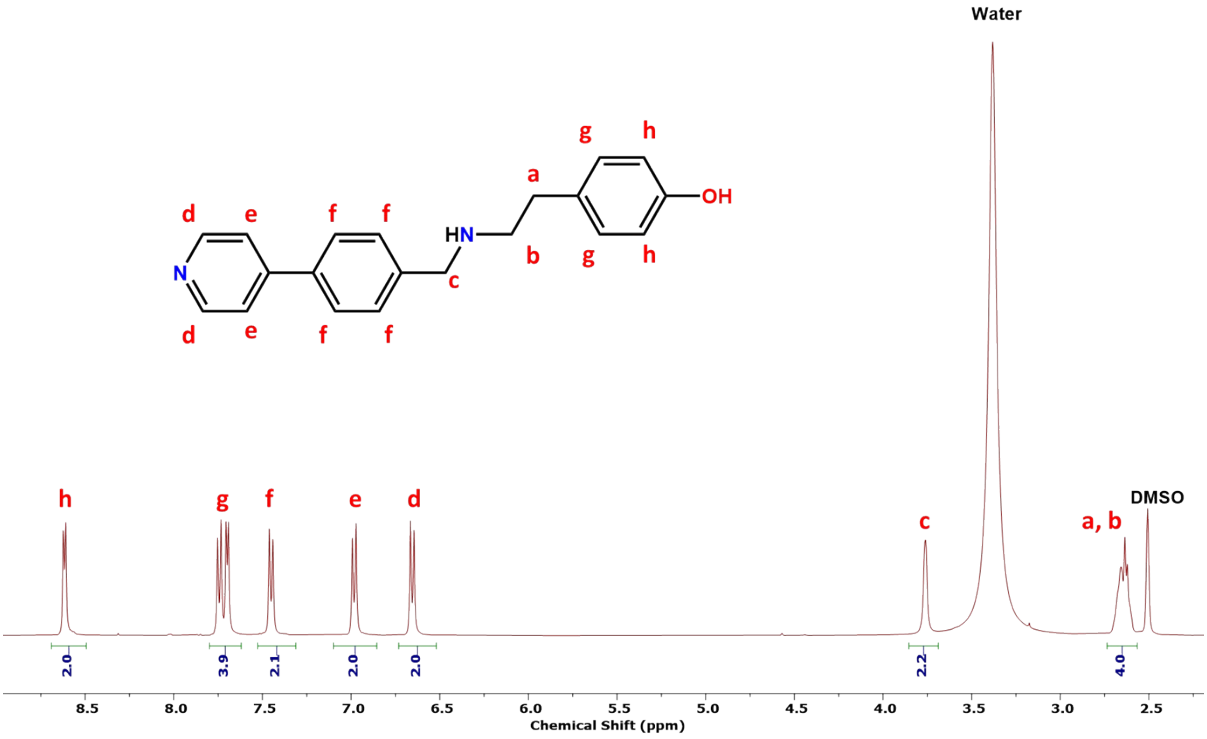

[$C_{20}H_{21}N_2O$][$Cr_7NiF_8(O_2C^tBu)_{16}$] (1E)

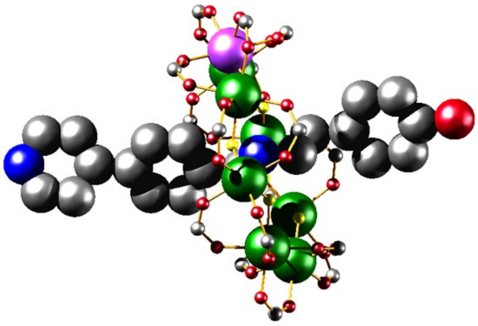

4-(2-((4-(pyridin-4-yl)benzyl)amino)ethyl)phenol (**E**) (2.50 g, 8.21 mmol, 1 eq) was added to 45 g of pivalic acid in an open Teflon flask alongside [$2NiCO_3\cdot3Ni(OH)_2\cdot4H_2O$] (2.00 g, 6.58 mmol, 0.8 eq) and $CrF_3\cdot4H_2O$ (12.00 g, 66.28 mmol, 8 eq) before heating to 160 °C for 24 h. The reaction was allowed to cool to room temperature before extracting the crude product in THF (100 mL) and solvent removed under a reduced pressure. The crude product purified by flash column chromatography (CH_2Cl_2: MeOH, 9:1) to give a green crystalline solid (2.38 g, 12 %). Elemental analysis (%) calcd. for $C_{100}H_{165}Cr_7F_8N_2NiO_{33}$: C 48.08, H 6.66, N 1.12; found: C 48.09, H 6.72, N 1.34. Positive ESI-MS (dissolved in THF, run in MeOH): m/z = 2520.6 [M+Na]$^+$.

[$C_{20}H_{21}N_2$][$Cr_7NiF_8(O_2C^tBu)_{16}$] (1A)

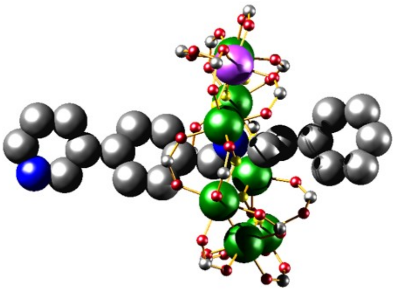

2-phenyl-N-(4-(pyridin-3-yl)benzyl)ethan-1-amine (**A**) (2.02 g, 7.00 mmol, 3 eq) was dissolved in 30 g of hot pivalic acid in an open Teflon flask before adding $CrF_3.4H_2O$ (3.00 g, 16.57 mmol, 7.1 eq) and stirring at 140 °C for 0.5 h. [$Ni_2(H_2O)(O_2CCMe_2)_4(HO_2CCMe_2)_4$] (1.33 g, 1.40 mmol, 0.6 eq) was then added slowly and the solution heated to 160 °C for 24 h. The reaction was allowed to cool to room temperature before addition of acetonitrile (100 mL) precipitating a green solid which was collected by filtration. The product was extracted in toluene (100 mL) and solvent removed under a reduced pressure. The crude product purified by flash column chromatography (CH_2Cl_2: EtOAc, 9:1) to give a green crystalline solid (3.84 g, 66 %). Elemental analysis (%) calcd. for $C_{100}H_{165}Cr_7F_8N_2NiO_{32}$: C 48.39, H 6.70, N 1.13; found: C 48.87, H 6.80, N 1.16. Positive ESI-MS (dissolved in THF, run in MeOH): m/z = 2504.6 [M+Na]$^+$.

$[C_{16}H_{21}N_2S][Cr_7NiF_8(O_2C^tBu)_{16}]$ (1B)

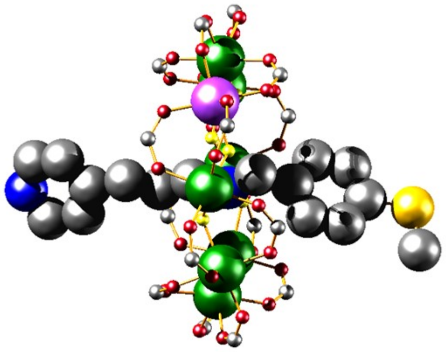

N-(4-(methylthio)benzyl)-3-(pyridin-4-yl)propan-1-amine (**B**) (0.64 g, 2.33 mmol, 1 eq) was dissolved in 30 g of hot pivalic acid in an open Teflon flask before adding $CrF_3.4H_2O$ (3.00 g, 16.57 mmol, 7.1 eq) and stirring at 140 °C for 0.5 h. $[Ni_2(H_2O)(O_2CCMe_2)_4(HO_2CCMe_2)_4]$ (0.60 g, 0.633 mmol, 0.27 eq) was then added slowly and the solution heated to 160 °C for 24 h. The reaction was allowed to cool to room temperature before addition of MeCN (100 mL) precipitating a green solid which was collected by filtration. The product was extracted in toluene (100 mL) and solvent removed under a reduced pressure. The crude product purified by flash column chromatography (CH_2Cl_2: EtOAc, 9:1) to give a green crystalline solid (1.88 g, 33 %) which was washed with cold acetone (10 mL). Elemental analysis (%) calcd. for $C_{102}H_{177}Cr_7F_8N_2NiO_{34}S$ (+2 acetone): C 47.44, H 6.91, N 1.08; found: C 47.61, H 7.07, N 1.04. Positive ESI-MS (dissolved in THF, run in MeOH): m/z = 2488.6 $[M+Na]^+$.

$[C_{25}H_{25}N_2O_2][Cr_7NiF_8(O_2C^tBu)_{16}]$ (1D)

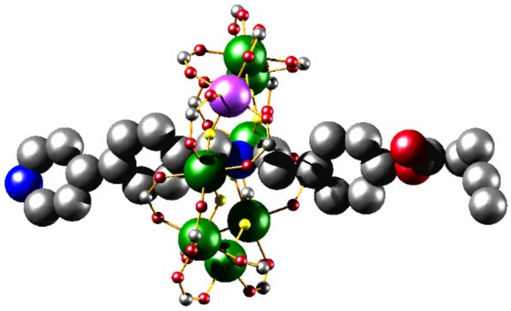

Rotaxane (**1E**) (0.51 g, 0.20 mmol) was dissolved in 50 mL dry THF alongside pentynoic acid (85.0 mg, 0.87 mmol), DMAP (0.311 mg, 2.55 mmol) and coupling agent DCC (0.32 g, 1.53 mmol). The reaction mixture was stirred under a nitrogen atmosphere for 16 h at room temperature. The solvent was removed under a reduced pressure and crude product purified by flash column chromatography ($CHCl_3$: EtOAc, 9:1) to give a green crystalline solid (0.37 g, 70 %). Elemental analysis (%) calcd. for $C_{105}H_{169}Cr_7F_8N_2NiO_{34}$: C 48.92, H 6.61, N 1.09; found: C 48.96, H 6.67, N 1.12. Positive ESI-MS (dissolved in THF, run in MeOH): m/z = 2600.6 $[M+Na]^+$.

[2(1A)$_6$]

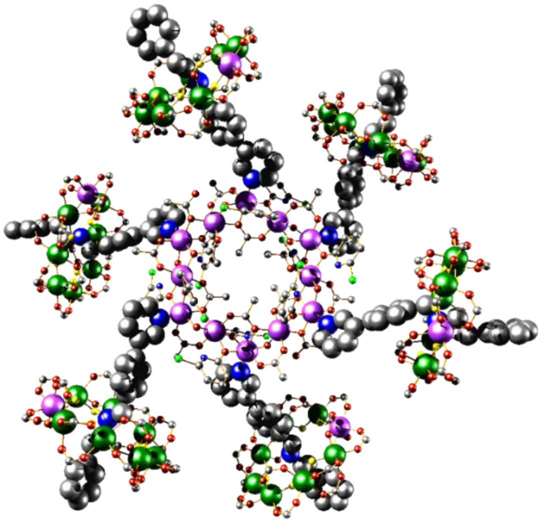

Compound **1A** (75.0 mg, 0.03 mmol, 6 eq) was dissolved in 5 mL of toluene and added to a suspension of compound **2** (17.6 mg, 0.005 mmol, 1 eq) in 5 mL of toluene. The resulting mixture was stirred for 10 minutes at 60 °C before filtering the resulting solution. The solvent was removed under a reduced pressure to yield a green powder which was recrystallized from vapour diffusion of MeCN into toluene. Small, green, well-defined crystals suitable for characterisation by single crystal XRD were obtained (40.5 mg, 45 %). Elemental analysis (%) calcd. for $C_{684}H_{1086}Cl_{12}Cr_{42}F_{48}N_{24}Ni_{18}O_{234}$: C 45.72, H 6.09, N 1.87; found: C 45.86, H 6.13, N 1.51.

[2(1B)$_6$]

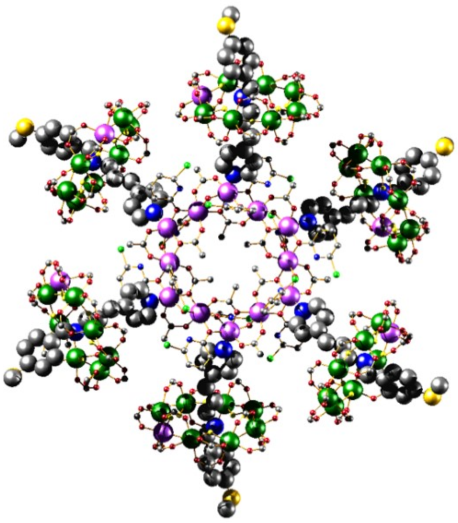

Compound **1B** (75.0 mg, 0.08 mmol, 6 eq) was dissolved in 5 mL of toluene and added to a suspension of compound **2** (17.7 mg, 0.005 mmol, 1 eq) in 5 mL of toluene. The resulting mixture was stirred for 10 minutes at 60 °C before filtering the resulting solution. The solvent was removed under a reduced pressure to yield a green powder which was recrystallized from vapour diffusion of MeCN into toluene. Small, green, well-defined crystals suitable for characterisation by single crystal XRD were obtained

(46.0 mg, 51 %). Elemental analysis (%) calcd. for $C_{660}H_{1086}Cl_{12}Cr_{42}F_{48}N_{24}Ni_{18}O_{234}S_6$: C 44.36, H 6.13, N 1.88; found: C 44.53, H 6.26, N 1.42.

[2(1C)$_6$]

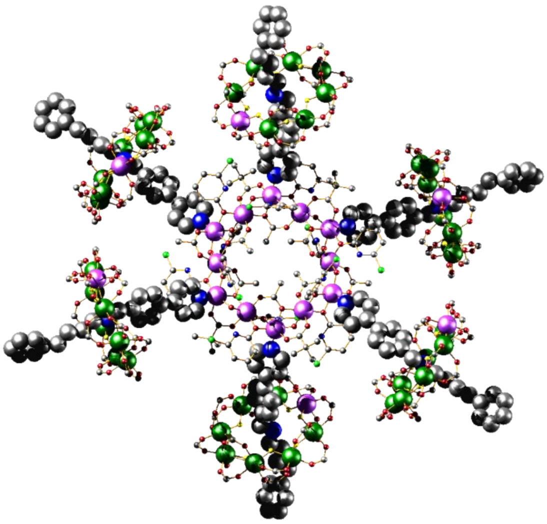

Compound **1C** (100.0 mg, 0.04 mmol, 6 eq) was dissolved in 5 mL of toluene and added to a suspension of compound **2** (23.2 mg, 0.007 mmol, 1 eq) in 5 mL of toluene. The resulting mixture was stirred for 10 minutes at 60 °C before filtering the resulting solution. The solvent was removed under a reduced pressure to yield a green powder which was recrystallized from vapour diffusion of MeCN into toluene. Small, green, well-defined crystals suitable for characterisation by single crystal XRD were obtained (49.1 mg, 40 %). Elemental analysis (%) calcd. for $C_{696}H_{1110}Cl_{12}Cr_{42}F_{48}N_{24}Ni_{18}O_{234}$: C 46.09, H 6.17, N 1.85; found: C 46.01, H 6.12, N 1.71.

[2(1D)$_6$]

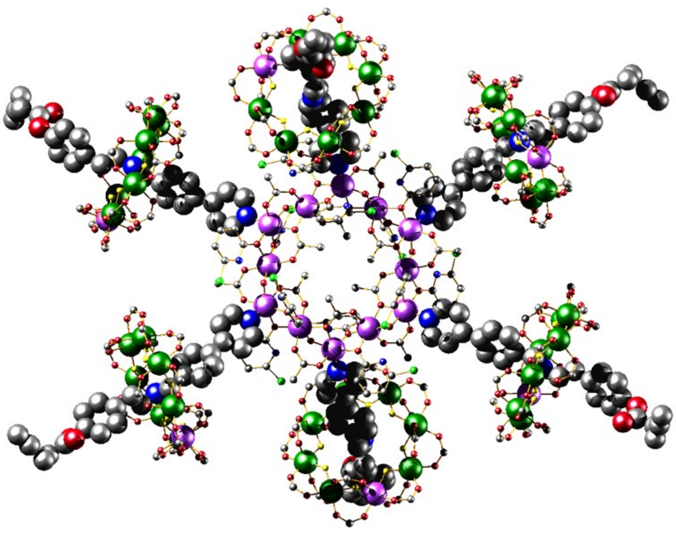

Compound **1D** (100.0 mg, 0.04 mmol, 6 eq) was dissolved in 5 mL of toluene and added to a suspension of compound **2** (22.6 mg, 0.006 mmol, 1 eq) in 5 mL of toluene. The resulting mixture was stirred for

10 minutes at 60 °C before filtering the resulting solution. The solvent was removed under a reduced pressure to yield a green powder which was recrystallized from vapour diffusion of MeCN into toluene. Small, green, well-defined crystals suitable for characterisation by single crystal XRD were obtained (59.0 mg, 49 %). Elemental analysis (%) calcd. for $C_{714}H_{1110}Cl_{12}Cr_{42}F_{48}N_{24}Ni_{18}O_{246}$: C 46.25, H 6.03, N 1.81; found: C 46.42, H 5.97, N 1.76.

Crystallography

Data collection

X-ray diffraction data for compound [**2**(**1A**)$_6$] were collected using a dual wavelength Rigaku FR-X rotating anode diffractometer using CuKα (λ = 1.54146 Å) radiation, equipped with an AFC-11 4-circle quarter-χ goniometer, VariMAX™ microfocus optics, a Hypix-6000HE detector and an Oxford Cryosystems Cryostream 700 plus nitrogen flow gas system, at a temperature of 150 K. Data for compound [**2**(**1B**)$_6$] were collected using a dual wavelength Rigaku FR-X rotating anode diffractometer using CuKα (λ = 1.54146 Å) radiation, equipped with an AFC-11 4-circle kappa goniometer, VariMAX™ microfocus optics, a Hypix-6000HE detector and an Oxford Cryosystems Cryostream 800 plus nitrogen flow gas system, at a temperature of 100 K. Data were collected using CrysAlisPro v42.[1] Data for compounds [**2**(**1C**)$_6$] and [**2**(**1D**)$_6$] were collected at Diamond Light Source beamline I19 using Zr-L edge wavelength (λ = 0.6889Å) radiation at a temperature of 100 K. Data were collected using GDA. All data were reduced using CrysAlisPro v42[1]. Absorption correction was performed using empirical methods (SCALE3 ABSPACK) based upon symmetry-equivalent reflections combined with measurements at different azimuthal angles.

Images of structures and electron density maps were rendered using POV-Ray 3.6.[4]

Refinement

Structure solution and refinement was carried out using Shelx-20XX, implemented through Olex2 v1.5.[X2,X3] Least-squared refinements against all F^2 values using ShelXL 2019/3. In all cases, the chromium and nickel in the Cr7Ni rings were assumed to be equally distributed, with each metal site constrained to have a 7:1 ratio of chromium to nickel, with the associated same coordinate and same atomic displacement parameter constraints. All organic moieties (pivalate, 6-chloro-2-hydroxopyridine, acetate and secondary ammonium cation threads) were refined to have similar 1,2- and 1,3- bond distances. Where modelled, disordered moieties were refined over two positions with the occupancy refined against independent free variable such that the sum of occupancy for each disordered associated pair equals 100%. Global similar neighbour atomic displacement parameter and rigid bond restraints were applied to the model. All hydrogens were modelled in idealised calculated positions, and in the case of the methyl hydrogens in idealised staggered geometry without refinement of the torsion angles. Diffuse solvent voids were modelled using an applied calculated solvent mask to account for the solvent contribution to the observed intensities.

[**2**(**1A**)$_6$]

Structure was solved using ShelXT 2018/2. In the case of the whole disordered Cr7Ni cluster (B/E), the inorganic chromium/nickel and fluoride rings were also refined to have similar 1,2- and 1,3- bond distances. There is evidence in the Ni12 cluster that there is substitution of 6-chloro-2-hydroxopyridine

for acetate, and in one circumstance this was clear enough that this substitutional disorder could be satisfactorily modelled.

[**2**(**1B**)$_6$]

Structure was solved using ShelXS. Additional fixed distance restraints were applied to the methylthioether group at the end of the ammonium cation threads. Flat restraints were also applied to the 6-chloro-2-hydroxopyridine moieties. Due to the low resolution of the data, all pivalate moieties were refined isotropically to retain a reasonable data to parameter ratio.

[**2**(**1C**)$_6$]

Structure was solved using ShelXT 2018/2. There is evidence in the Ni_{12} cluster that there is substitution of bridging acetate with pivalate, which was modelled as 100% pivalate. Additional 1,2- and 1,3- same distance restraints were applied to all equivalent bonds across the 6-chloro-2-hydroxopyridine moieties, rather than just the same bonds between moieties, in order to refine a suitable geometry. Flat restraints were applied to the terminal phenyl moieties and the neighbouring methylene carbon of the ammonium cation thread.

[**2**(**1D**)$_6$]

Structure was solved using ShelXT 2018/2. Whole disorder of the central Ni12 cluster and bound ammonium cation threads ($2D_6$) was identified and modelled over two positions against a single occupancy free variable. Additional 1,2- and 1,3- same distance restraints were applied to all equivalent bonds across the disordered moieties, rather than just the same bonds between moieties, to refine a suitable geometry. Where necessary, fixed distance and flat restraints were also applied. Strong similar neighbour atomic displacement parameter and rigid bond restraints were applied to the disordered moiety. Due to the low resolution of the data, and extensive disorder of the $2D_6$ moiety, all pivalate moieties were refined isotropically to retain a reasonable data to parameter ratio. Legacy restraints are also included in the instructions file from initial refinements of the disordered moieties as residues; these were not active during the final refinement steps.

Table S1. Crystallographic and refinement parameters for [**2**(**1A**)$_6$], [**2**(**1B**)$_6$], [**2**(**1C**)$_6$] and [**2**(**1D**)$_6$]

Compound	[**2**(**1A**)$_6$]	[**2**(**1B**)$_6$]
Identification code	LREPW391	rrepw297
Empirical formula	$C_{675.51}H_{1072}Cl_{9.17}Cr_{42}F_{48}N_{21.17}Ni_{18}O_{236.83}$	$C_{660}H_{1074}Cl_{12}Cr_{42}F_{48}N_{24}Ni_{18}O_{234}S_6$
Formula weight	17757.12	17859.91
Temperature/K	150.03(10)	100.0(4)
Crystal system	triclinic	triclinic
Space group	P-1	P-1
a/Å	22.9954(8)	30.4038(11)
b/Å	34.9940(10)	32.5701(12)
c/Å	37.8756(10)	33.5359(13)
α/°	76.494(2)	64.809(4)
β/°	74.969(3)	75.396(3)
γ/°	77.185(3)	78.083(3)
Volume/Å^3	28196.6(16)	28893(2)
Z	1	1
ρ_{calc} g/cm^3	1.046	1.026
μ/mm^{-1}	4.250	4.306
F(000)	9268.0	9318.0
Crystal size/mm^3	0.521 × 0.232 × 0.171	0.281 × 0.12 × 0.054
Radiation	Cu Kα (λ = 1.54184)	Cu Kα (λ = 1.54184)
2Θ range for data collection/°	3.926 to 117.868	2.962 to 76.882
Index ranges	-21 ≤ h ≤ 25, -38 ≤ k ≤ 38, -42 ≤ l ≤ 42	-24 ≤ h ≤ 24, -26 ≤ k ≤ 26, -26 ≤ l ≤ 27
Reflections collected	314726	147207
Independent reflections	80098 [R_{int} = 0.1223, R_{sigma} = 0.0969]	31738 [Rint = 0.1071, Rsigma = 0.0931]
Data/restraints/parameters	80098/51502/5935	31738/23682/3463
Goodness-of-fit on F^2	0.964	1.404
Final *R* indexes [I > =2σ(I)]	R_1 = 0.0827, wR_2 = 0.2356	R1 = 0.1270, wR2 = 0.3628
Final *R* indexes [all data]	R_1 = 0.1618, wR_2 = 0.2921	R1 = 0.1880, wR2 = 0.4048
Largest diff. peak/hole / e Å^{-3}	0.78/-0.62	0.71/-0.53

Table S1 continued

Compound	[**2**(**1C**)$_6$]	[**2**(**1D**)$_6$]
Identification code	tb0104_c3_col1_	tb095_c2_col11_0
Empirical formula	$C_{708}H_{1122}Cl_{12}Cr_{42}F_{48}N_{24}Ni_{18}O_{234}$	$C_{714}H_{1098}Cl_{12}Cr_{42}F_{48}N_{24}Ni_{18}O_{246}$
Formula weight	18292.41	18532.28
Temperature/K	100.00(10)	100.0(2)
Crystal system	monoclinic	triclinic
Space group	P2/n	P-1
a/Å	43.4575(4)	19.5330(4)
b/Å	19.0687(3)	41.0545(7)
c/Å	68.7276(6)	41.2913(9)
α/°	90	116.6532(19)
β/°	91.4880(10)	97.2999(17)
γ/°	90	97.6664(16)
Volume/Å^3	56933.8(12)	28668.7(11)
Z	2	1
ρ_{calc} g/cm^3	1.067	1.073
μ/mm^{-1}	0.708	0.704
F(000)	19116.0	9666.0
Crystal size/mm^3	0.06 × 0.06 × 0.05	0.03 × 0.025 × 0.025
Radiation	synchrotron (λ = 0.6889)	synchrotron (λ = 0.6889)
2*Θ* range for data collection/°	2.966 to 37.196	2.948 to 38.684
Index ranges	-40 ≤ h ≤ 40, -17 ≤ k ≤ 17, -63 ≤ l ≤ 63	-18 ≤ h ≤ 18, -39 ≤ k ≤ 39, -39 ≤ l ≤ 39
Reflections collected	385404	127301
Independent reflections	47320 [R_{int} = 0.1380, R_{sigma} = 0.0867]	52206 [R_{int} = 0.0742, R_{sigma} = 0.1115]
Data/restraints/parameters	47320/29321/4861	52206/33252/5282
Goodness-of-fit on F^2	1.220	1.312
Final *R* indexes [I > =2$\sigma(I)$]	R_1 = 0.1096, wR_2 = 0.3228	R_1 = 0.1416, wR_2 = 0.3796
Final *R* indexes [all data]	R_1 = 0.1383, wR_2 = 0.3495	R_1 = 0.1968, wR_2 = 0.4260
Largest diff. peak/hole / e Å^{-3}	0.94/-0.38	0.92/-0.49

Table S2. Structural parameters within the [7]rotaxanes [**2**(**1X**)$_6$] in the crystal structure (CS) and in the optimised conformer (OC) used for MD simulations. Estimated standard deviations on bonds < 0.01 Å and on angles < 0.1 °.

Thread	**A**		**B**		**C**		**D**	
	CS	OC	CS	OC	CS	OC	CS*	OC
{Cr_7Ni} centroid – Ni(Ni_{12}}/ Å	10.51<br>10.44<br>10.76	10.23<br>10.23<br>10.23<br>10.24<br>10.24<br>10.26	9.48<br>9.52<br>9.60	9.13<br>9.14<br>9.14<br>9.15<br>9.16<br>9.17	11.24<br>11.33<br>11.34	10.45<br>10.45<br>10.45<br>10.45<br>10.45<br>10.46	11.12<br>11.15<br>11.19	10.23<br>10.23<br>10.23<br>10.23<br>10.23<br>10.24
{Cr_7Ni} centroid – {Cr_7Ni} centroid/ Å	31.78<br>31.95<br>32.89	25.52<br>25.99<br>26.15	30.28<br>30.51<br>30.70	28.07<br>28.30<br>28.34	32.38<br>34.12<br>34.45	31.05<br>33.34<br>31.35	31.86<br>34.01<br>34.10	30.08<br>31.02<br>31.05
Diameter of [7]rotaxane/ Å	43.60<br>43.94<br>44.54	33.45<br>33.97<br>34.20	45.16<br>45.52<br>45.80	34.49<br>34.73<br>36.42<br>36.73<br>37.07	49.46<br>51.52<br>51.74	48.19<br>48.53<br>48.57	55.30<br>55.51<br>57.79	58.45<br>58.76<br>58.76
Angle between mean planes of **1X** and **2** /°	62.4<br>78.4<br>79.7	57.3<br>59.3<br>58.2<br>86.7<br>88.5<br>89.3	66.1<br>70.5<br>75.0	17.7<br>18.5<br>88.7<br>88.8<br>89.3	35.7<br>79.6<br>89.2	34.0<br>36.2<br>36.5<br>67.8<br>70.1<br>70.4	29.4<br>80.9<br>87.0	47.5<br>49.5<br>50.0<br>55.3<br>57.3<br>57.7
Angle of thread to mean plane of **2** /°	32.2<br>35.8<br>41.0	33.2<br>33.5<br>34.6<br>34.9<br>36.6<br>37.0	35.0<br>35.3<br>35.6	33.1<br>33.8<br>34.5<br>35.2<br>36.7<br>37.2	34.7<br>35.5<br>37.3	33.4<br>33.5<br>34.8<br>34.9<br>37.0<br>37.0	31.7<br>35.7<br>36.3	33.5<br>33.5<br>34.9<br>34.9<br>37.0<br>37.0

* Measurements made using major part of disordered crystal structure.

a.

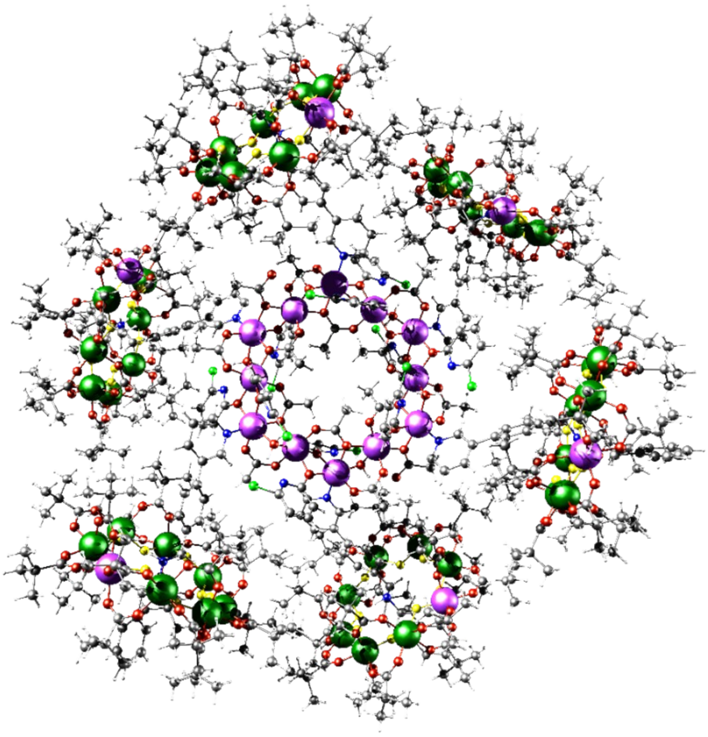

b.

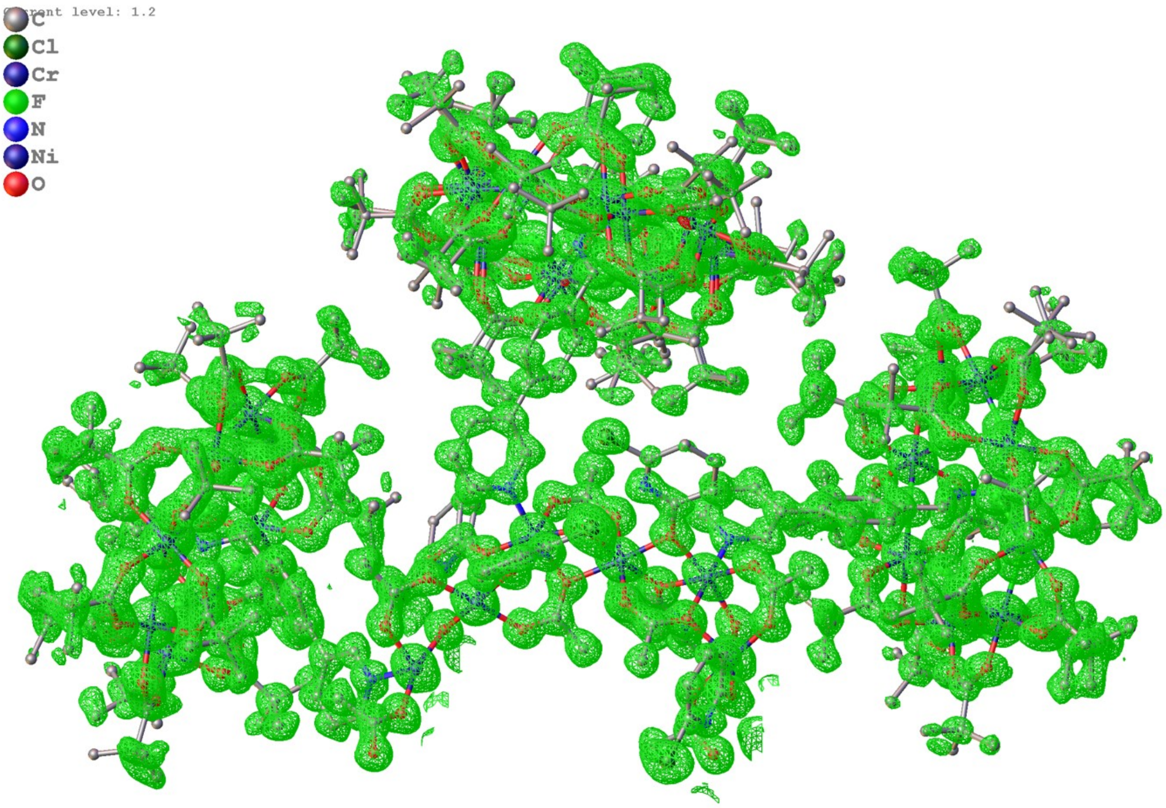

Figure S1. a. Single crystal XRD structure of [**2**(**1A**)$_6$]. Colour scheme: Cr dark green, Ni lilac, O red, N blue, C grey, F yellow, Cl bright green. The Ni sites in the {Cr_7Ni} rings are disordered over multiple positions. Disorder has been omitted for clarity. b. Electron density map of the asymmetric unit of [**2**(**1A**)$_6$].

a.

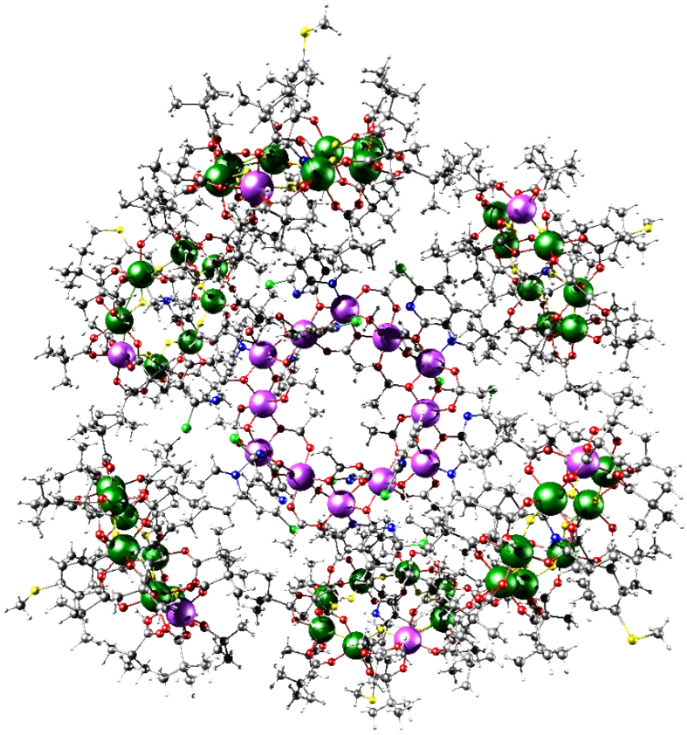

b.

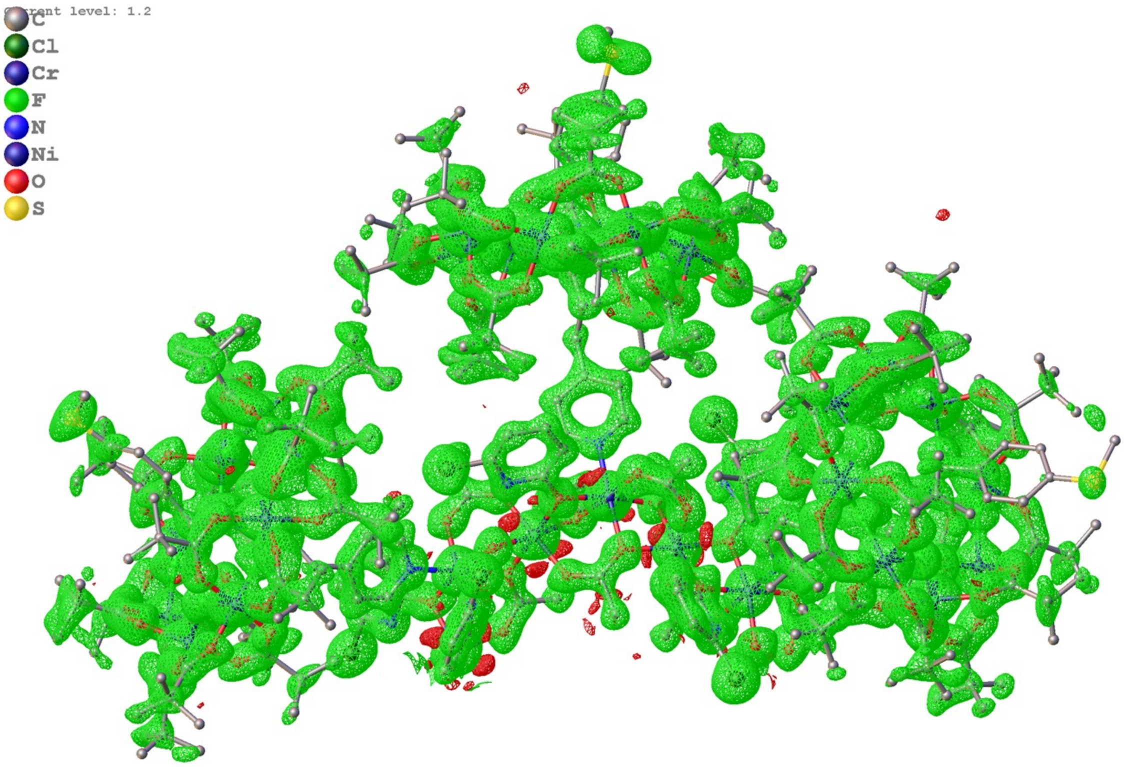

Figure S2. Single crystal XRD structure of [**2**(**1B**)$_6$]. Colour scheme as in Figure S1; S gold. The Ni sites in the {Cr_7Ni} rings are disordered over multiple positions. Disorder has been omitted for clarity. b. Electron density map of the asymmetric unit of [**2**(**1B**)$_6$].

a.

b.

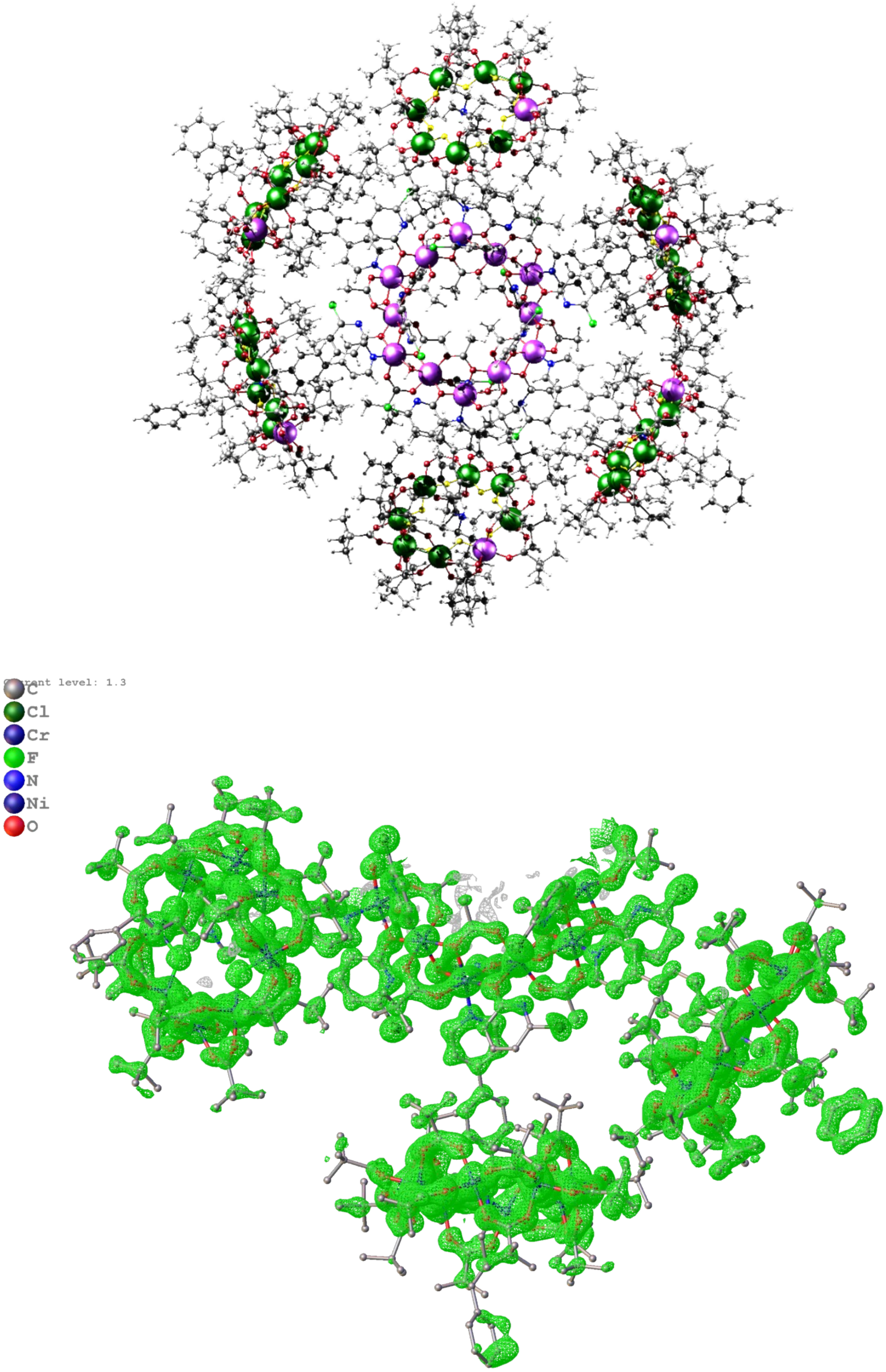

Figure S3. a. Single crystal XRD structure of [**2**(**1C**)$_6$]. Colour scheme as in Figure S1. The Ni sites in the {Cr$_7$Ni} rings are disordered over multiple positions. Disorder has been omitted for clarity. b. Electron density map of the asymmetric unit of [**2**(**1C**)$_6$].

a.

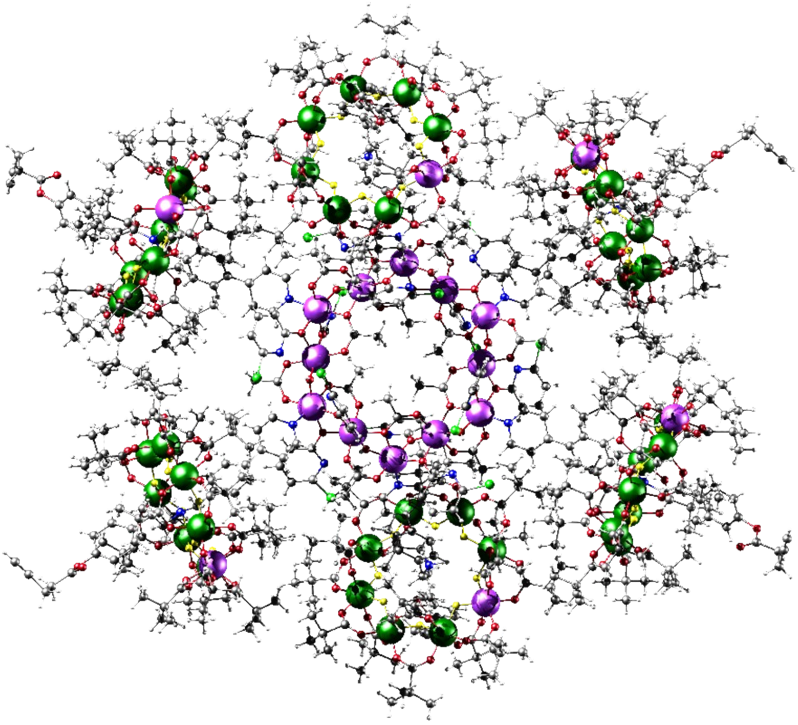

b.

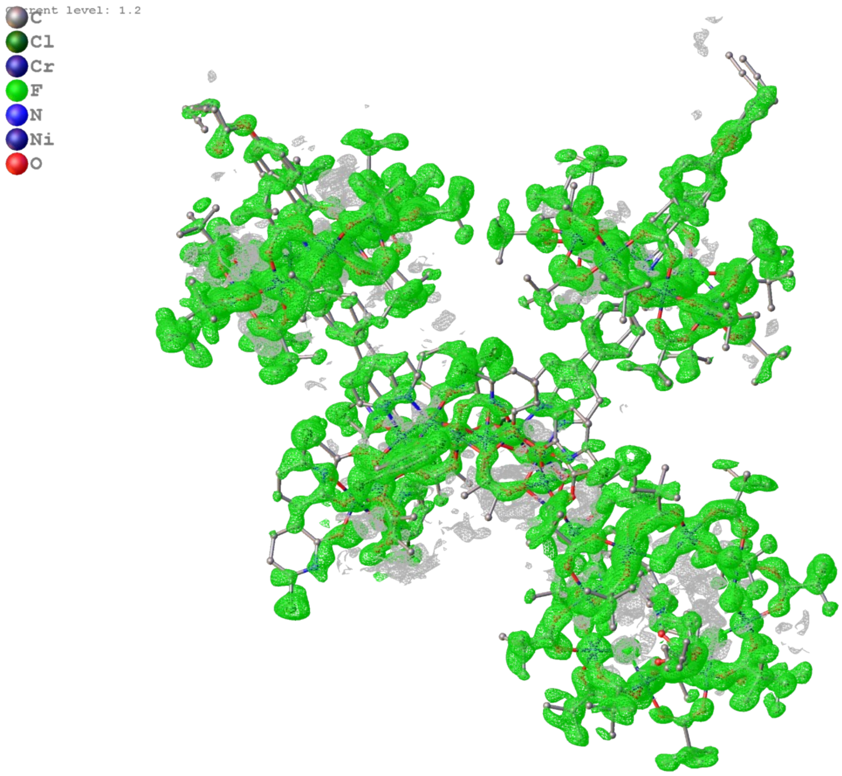

Figure S4. a. Single crystal XRD structure of [**2**(**1D**)$_6$]. Colour scheme as in Figure S1. The Ni sites in the {Cr_7Ni} rings are disordered over multiple positions. Disorder has been omitted for clarity. b. Electron density map of the asymmetric unit of [**2**(**1D**)$_6$].

Small angle X-ray scattering

SAXS measurements were performed on a HECUS SAXS/GISAXS instrument equipped with XENOCS micro focus CuK_α (λ = 1.5407 Å) source equipped with Montel optics and the diffracted X-rays collected with a Dectris Pilatus 100 K 2D detector. Samples were dissolved in toluene contained in borosilicate capillaries with diameter of 1 mm and wall thickness of 10 µm. Silver behenate was used for calibration of the instrument before every collection. Pure toluene collection was performed with identical conditions as the samples to allow consistent subtraction. Sample collections typically took 10000 seconds. All experimental data are the sum of the 2D radial distribution of the small angle X-ray diffraction converted to a 1D line graph. Irena SAS/SANS routines in Wavemetrics Igor Pro have been used for calibration,[5] data conversion and subsequent analysis.

The analysis involved subtracting the solvent contribution from the sample and solvent data before employing routines in Irena for the analysis. Pair distance distribution functions provided a reliable, simple and reproducible means for investigating the molecular sizes. The corrected data was analysed using the Moores method.[5] Initially, the approximate size is determined and then function fitted to a region between large aggregate signals (small angles) and the statistically insignificant data at high angles. Fitting was repeated until a steady maximum size was achieved.

Atomistic molecular dynamics simulations

All MD simulations were performed at 298 K in a toluene solution to relax the most promising constructed structures, and to obtain more realistic ensemble structural averages from which the experimental SAXS and corresponding pair pair P(r) distribution is made are made comparison to. All simulations were performed using Gromacs 2016.4 molecular dynamics package[6,7] The parameterization of the systems was adapted to the General Amber Forcefield.[8] A single atomistic structure of each conformer in a solution of toluene at in a cubic box size of 10.00 nm^3 was set up.

To prepare the systems prior to performing full production run simulation, energy minimisation was performed using the conjugate gradient algorithm. The minimised system was followed by a short NPT ensemble simulation for 10 ns (T = 298 K and p = 1.01325 bar) using the Nosé–Hoover thermostat[9] and the Parrinello–Rahman barostat.[10] The particle-mesh Ewald method[11] was used to compute the electrostatics. Periodic boundary conditions were used and the time step for Integration was fixed at 1fs with the neighbour list updated every 10 fs.

Small Angle X-Ray Scattering (SAXS) was calculated on selected obviously different conformers of each rotaxane, subsequently the choice for the structure to be set up for Molecular Dynamics simulation to be compared directly to SAXS data from experiments. Scattering factors were taken from computed X-ray scattering factors from Hartree-Fock calculations.[12] The SAXS box used for all calculated profiles was 100 nm with a X-ray wavelength of 0.154209 nm. SAXS profiles were plotted as a natural log of intensity ln I(q)(a.u.) against the scattering vector, q (Å). SAXS calculation were done on the whole structure alongside the various different rotated conformers.

Irena SAS/SANS routines in wavemetrics in Igor Pro[5] have been used to calculate the pair distribution function from the SAXS data obtained from MD. The Pair distribution function using routines in Irena has enables us to investigate and compare the molecular size to experimental data. Moores method[5]

was used to approximate the size and then fitted to a function in the region of large aggregate signals (small angles) and the statistically insignificant data at high angles. The fitting was repeated for all data sets until a maximum size was achieved.

The SAXS profile and P(r) functions were all visually inspected and compared directly to corresponding experimental data.

To assess whether the disparity of the preliminary SAXS predictions was a result of disorder in the crystal structure, or possible detachment of the metal rings in solution, we have constructed and analysed the SAXS spectra of a comprehensive range of possible rotaxane model structures.

In total, 100 unique initial static conformations of the four rotaxanes (64 for [**2**(**1A**)$_6$], 4 for [**2**(**1B**)$_6$], 16 for [**2**(**1C**)$_6$] and 16 for [**2**(**1D**)$_6$]) were assessed for viability. These model structures were constructed from three components: the {Ni_{12}} core (**2**), {Cr_7Ni} anionic rings (**1**) and protonated threads (**A**, **B**, **C** and **D**). The initial structural conformations of the {Ni_{12}} core and the {Cr_7Ni} ring components were based on the XRD structures; however to facilitate construction of the full [7]rotaxane, each of the three main components was independently optimised in the gas phase using density functional theory (DFT) using the B3LYP exchange-correlation functional and a 6-31G(d) basis set on HCNOF atoms, and a LANL2 effective core potential/double zeta basis set for Cl, Cr(III) and Ni(II); all metals were high spin and ferromagnetically coupled within the **2** and anti-ferromagnetically coupled within the Cr_7Ni rings. Since full optimisation of the model [7]rotaxane at this level was not possible, in order to aid selection of feasible models and initiate molecular dynamics simulations of the structures, a primitive distance-based scoring function was used to assess the number and severity of the bad-contacts (short non-bond interactions due to close proximity or overlap of functional groups) in each model. This was subsequently used to make minor rotational and translational adjustments to the relative orientations of the thread, metal ring, pivalate ligands and the {Ni_{12}} core before simulation.

Figure S5 summarises the four threads and the rotational conformations of each thread used to construct the model rotaxanes. It may be noted that for [**2**(**1A**)$_6$], rotamers Q and R are 0.8 kJ mol^{-1} higher in energy than P and S at the B3LYP/6-31G(d) level, but for the other threads the rotamers are all energetically degenerate. In the XRD only conformers P were evident for [**2**(**1A**)$_6$], [**2**(**1B**)$_6$] and [**2**(**1C**)$_6$], and the Q conformer for [**2**(**1D**)$_6$] - nevertheless the resolution of the conformations of these structures was poor. Figure S10 illustrates how the meta-substituted pyridyl threads of [**2**(**1A**)$_6$] may be oriented in either the X or E-thread directions, and/or that different conformers may alternate around the {Ni_{12}} ring; the symmetry of threads [**2**(**1B**)$_6$], [**2**(**1C**)$_6$] and [**2**(**1D**)$_6$] does limit the number of conformations since the X and E orientations are equivalent to P/Q or R/S rotamers.

a

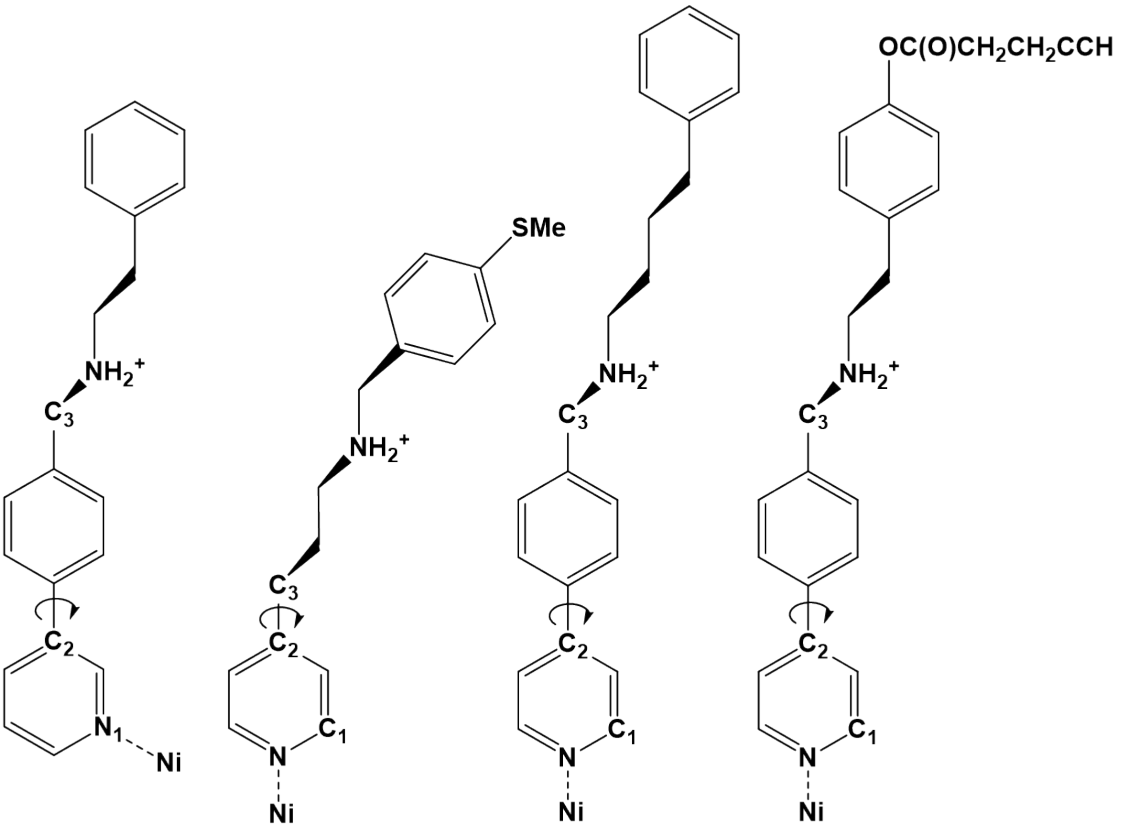

A **B** **C** **D**

b $\tau(N_1\text{-}C_2\text{-}C_3\text{-}NH_2)$ or $\tau(C_1\text{-}C_2\text{-}C_3\text{-}NH_2)$

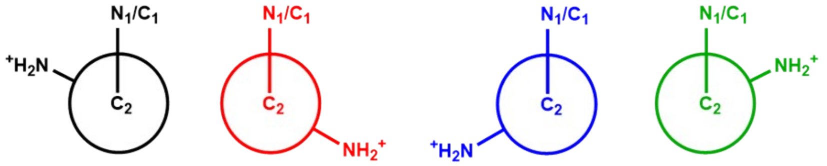

P **Q** **R** **S**

Figure S5. a, The four threads **A**, **B**, **C** and **D** used in the synthesis of [7]rotaxanes [**2**(**1A**)$_6$], [**2**(**1B**)$_6$], [**2**(**1C**)$_6$] and [**2**(**1D**)$_6$] respectively (the pyridyl head groups are shown coordinating to Ni ions; and b, Newman projections of the $\tau(N_1/C_1\text{-}C_2\text{-}C_3\text{-}NH_2^+)$ dihedral angle of the four rotational conformers considered, P, Q, R and S ([**2**(**1B**)$_6$] only has P and Q conformations).

The nomenclature of the Newman projections defining the rotational conformers of the thread has been used to define the [7]rotaxane conformers as displayed in Figures S6 – S9. Each conformer contains coordinated threads where the pyridyl group binding alternates above and below the plane of **2**. Each set of alternate threads is defined by a Newman projection which corresponds to the rotational conformer of the thread. For example, conformer PQ contains three coordinated threads containing rotamer P and three containing rotamer Q (Figure S7).

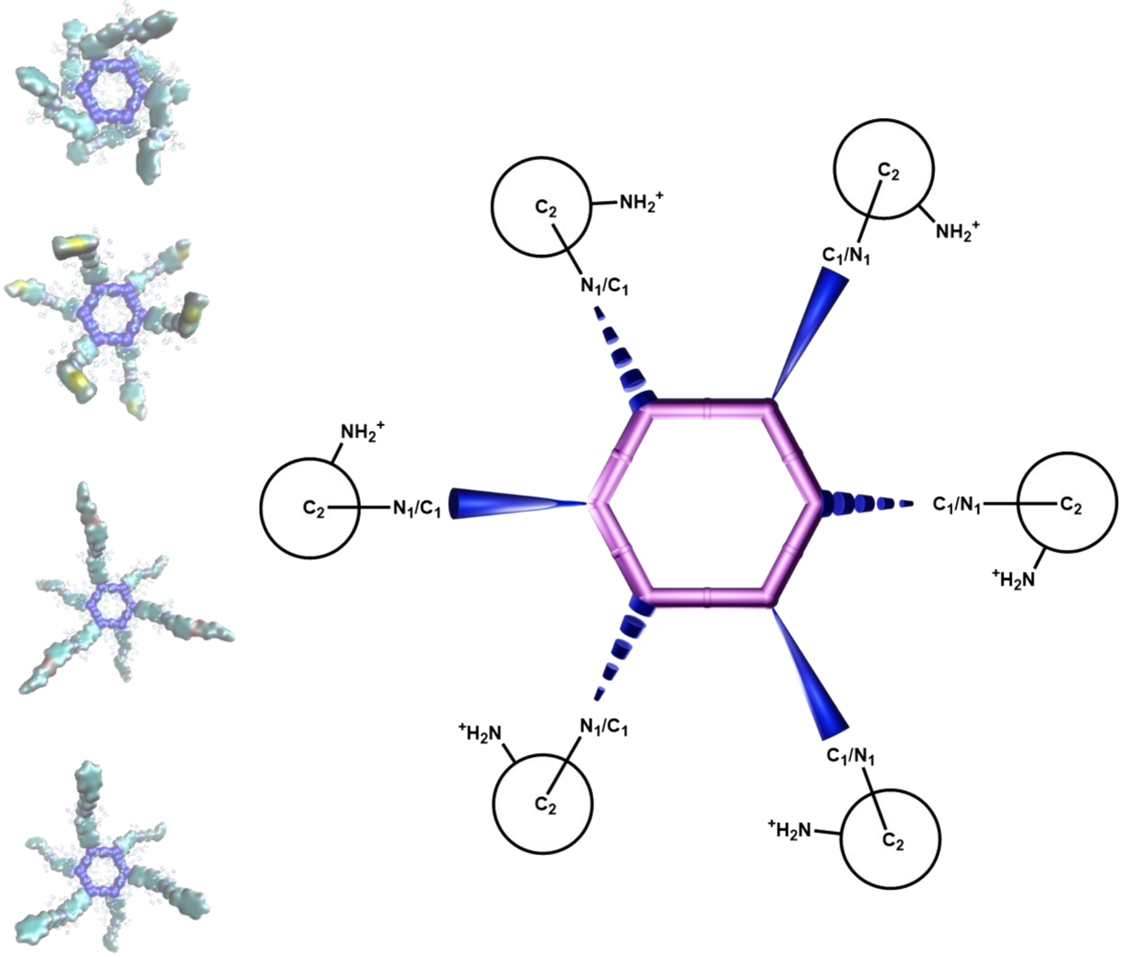

Figure S6. Diagram displaying thread rotational conformers present in conformers PP and PT alongside images of conformers used to obtain SAXS spectra for molecules [**2**(**1A**)$_6$], [**2**(**1B**)$_6$], [**2**(**1C**)$_6$] and [**2**(**1D**)$_6$]. Conformers PP and PT differ only in the direction of the ammonium thread with respect to the plane of **2**. Identity of Newman projections are as defined in Figure S5.

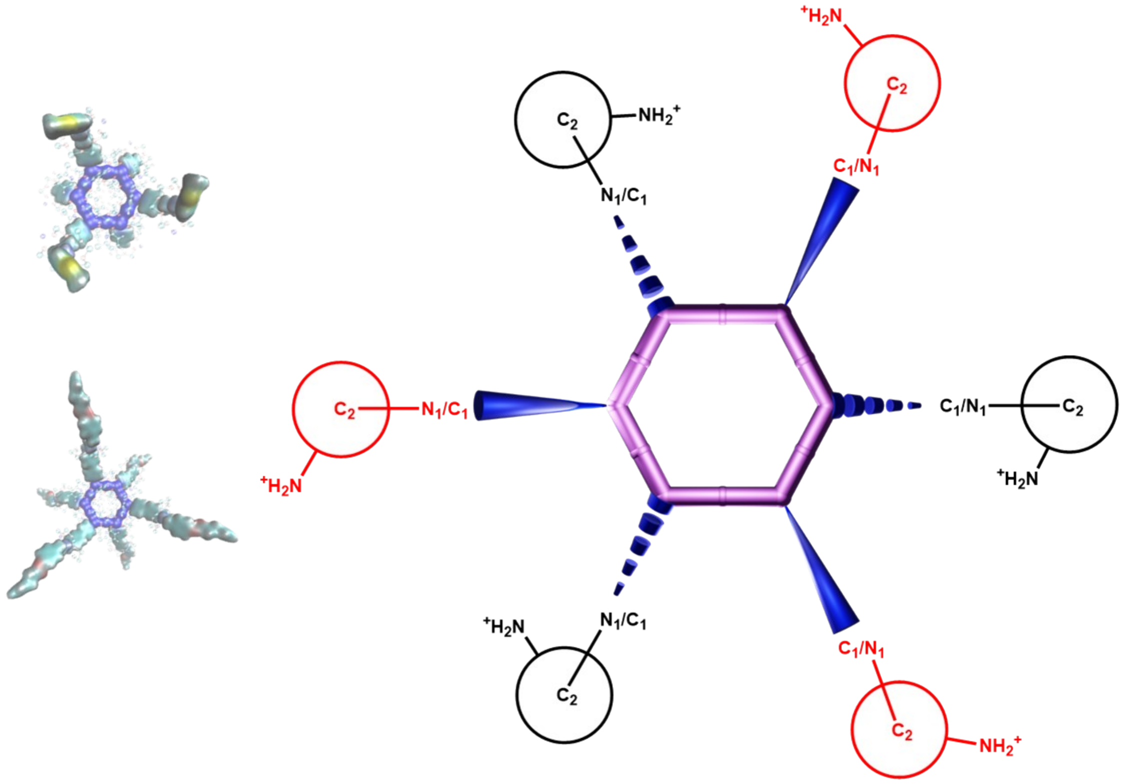

Figure S7. Diagram displaying thread rotational conformers present in conformer QP alongside images of conformers used to obtain SAXS spectra for molecules [**2**(**1B**)$_6$], and [**2**(**1C**)$_6$]. Identity of Newman projections are as defined in Figure S5.

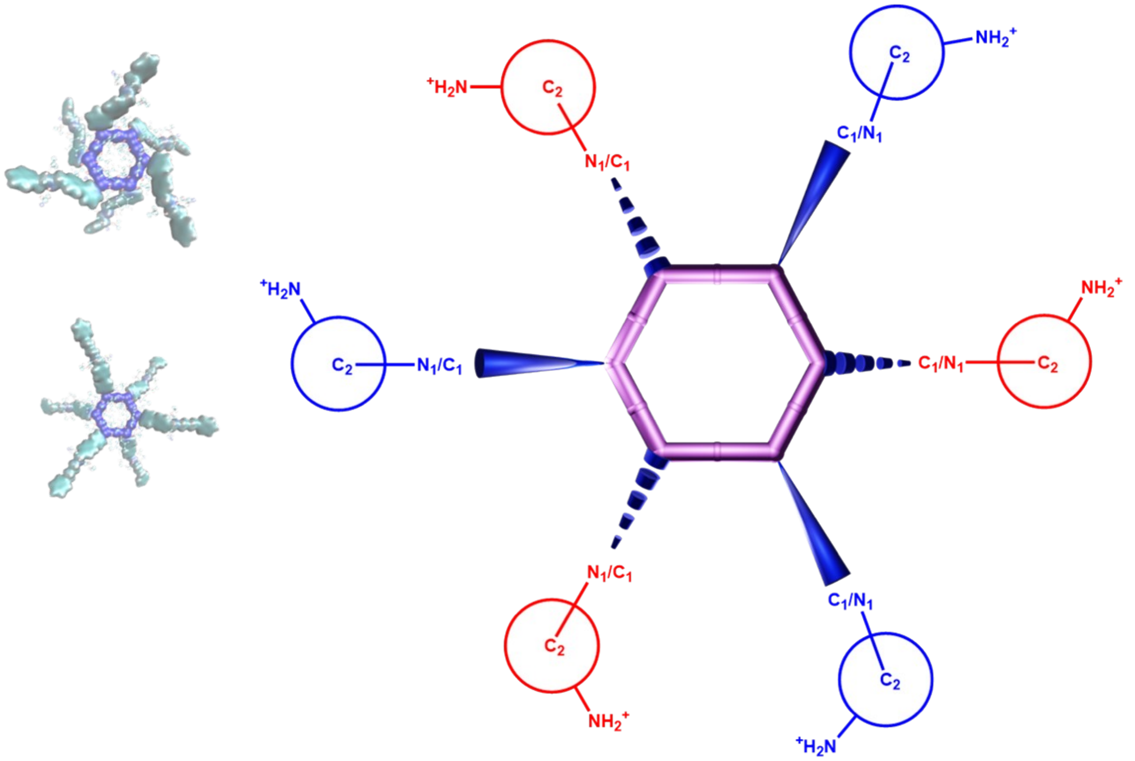

Figure S8. Diagram displaying thread rotational conformers present in conformer RQ alongside images of conformers used to obtain SAXS spectra for molecules [**2**(**1A**)$_6$] and [**2**(**1D**)$_6$]. Identity of Newman projections are as defined in Figure S5.

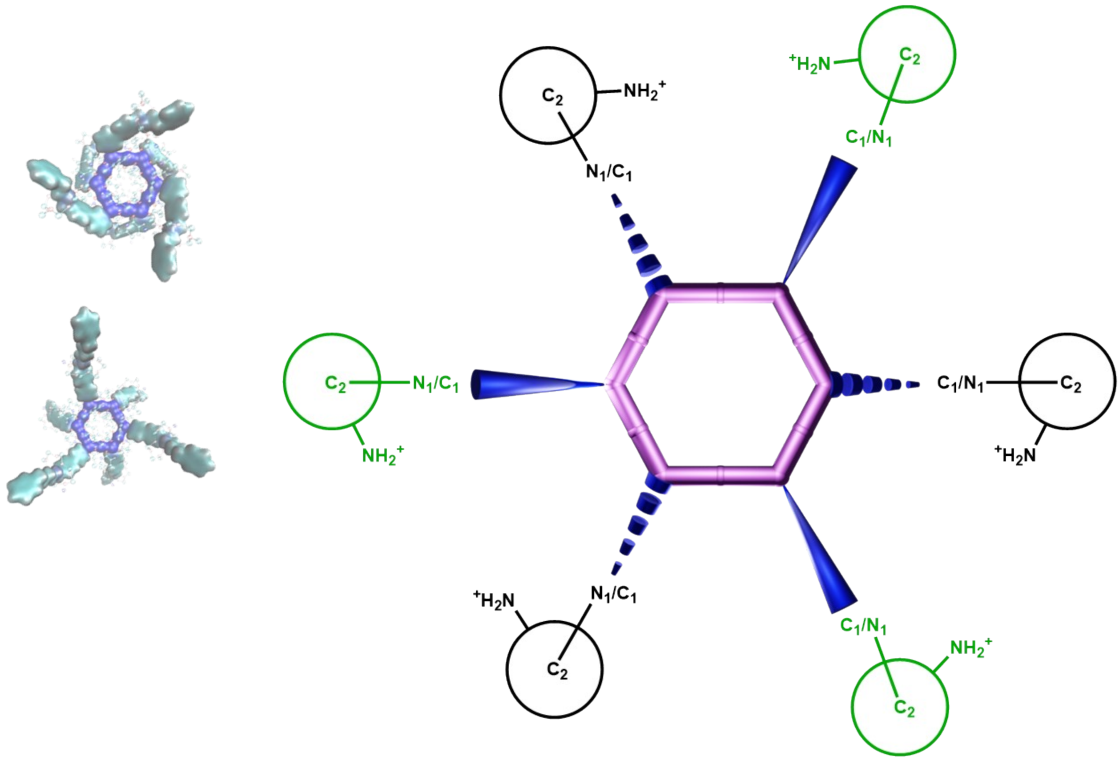

Figure S9. Diagram displaying thread rotational conformers present in conformer SP alongside images of conformers used to obtain SAXS spectra for molecules [**2**(**1A**)$_6$] and [**2**(**1D**)$_6$]. Identity of Newman projections are as defined in Figure S5.

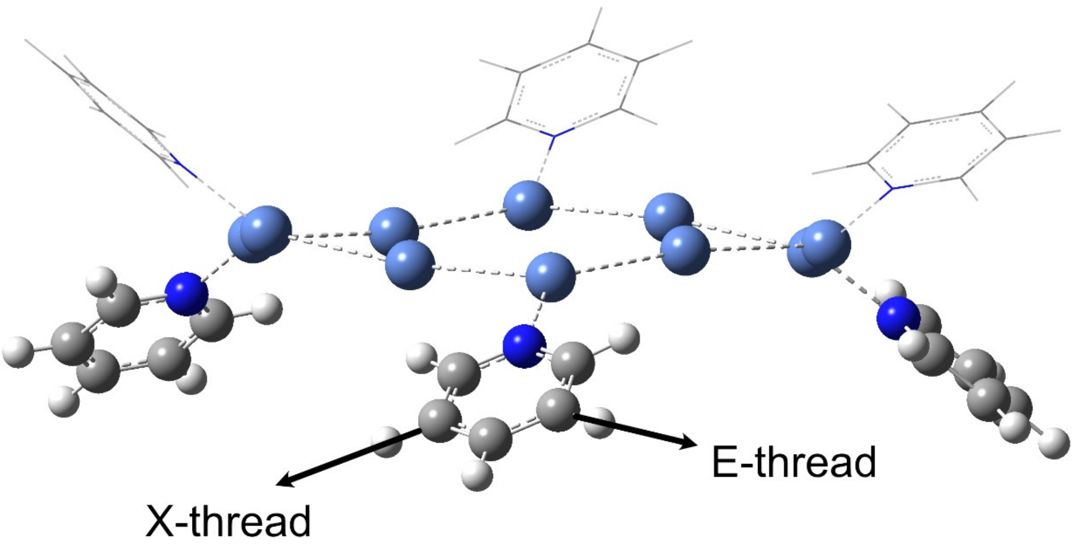

Figure S10. The $\{Ni_{12}\}$ ring and six pyridine ligands which are substituted by the threads (all other species are omitted for clarity). X- and E- orientations are possible for the meta-substituent of the pyridine head-group in [**2**(**1A**)$_6$]. Alternating pyridine groups (ball-and-stick or wireframe) can be substituted in [**2**(**1B**)$_6$] with different thread rotamers.

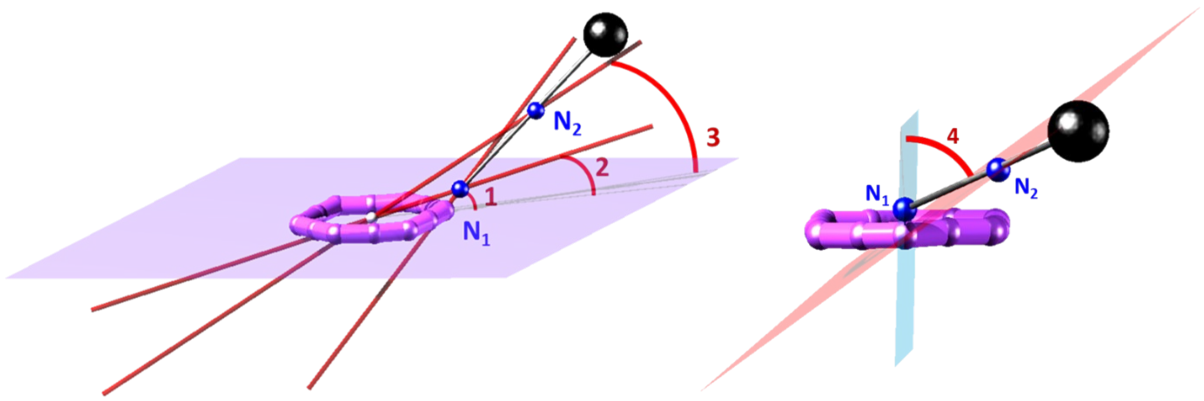

Figure S11. Diagram describing angles 1, 2, 3 and 4 used to compare [7]rotaxane conformers.

To compare conformers PP, PT, QP, RQ and SP to one another, angles were measured using the plane of **2** (Figure S11). Angle 1 is defined by the angle of thread coordination by calculating the angle that the Ni···N$_1$ bond makes with the plane of **2** (where N$_1$ = pyridyl nitrogen). Angles 2 and 3 correspond to the N$_1$···{**2**}$_{centroid}$···{**2**}$_{plane}$ and N$_2$···{**2**}$_{centroid}$···{**2**}$_{plane}$ angles respectively (where N$_2$ = ammonium nitrogen). Angle 4 is defined by the angle measured between planes created through atoms N$_1$···{**2**}$_{centroid}$···Ni and N$_2$···{**2**}$_{centroid}$···Ni. The angles associated with conformers of each molecule have been displayed alongside the crystal structure (CS) measurements (Table S3).

Table S3. Angles measured on the SAXS conformers and crystal structures of [**2**(**1A**)$_6$] – [**2**(**1D**)$_6$].

Compound	Conformer	Angle 1: Coordination angles N_1···Ni···{**2**}$_{plane}$ /°	Angle 2: N_1···{**2**}$_{centroid}$···{**2**}$_{plane}$ /°	Angle 3: N_2···{**2**}$_{centroid}$···{**2**}$_{plane}$ /°	Angle 4: Angle between planes of N_1···{**2**}$_{centroid}$···Ni and N_2···{**2**}$_{centroid}$···Ni /°
[**2**(**1A**)$_6$]	PP	34.6, 33.5, 36.6, 34.9, 33.2, 37.0	11.3, 10.3, 10.0, 11.2, 10.4, 9.90	20.0, 27.9, 17.4, 30.8, 17.4, 27.9	42.2, 30.6, 43.6, 28.9, 43.9, 30.3
	PT	33.8, 37.0, 35.1, 33.5, 37.3, 34.8	10.1, 11.3, 10.5, 10.0, 11.4, 10.4	6.59, 15.0, 7.47, 14.4, 5.24, 74.7	84.6, 80.2, 87.0, 80.9, 85.0, 77.8
	RQ	36.6, 34.5, 33.1, 36.6, 34.5, 33.2	11.2, 10.3, 9.90, 11.2, 10.3, 9.90	23.3, 19.5, 20.6, 22.3, 20.7, 19.6	34.8, 38.1, 36.2, 36.1, 36.5, 37.5
	SP	36.6, 34.6, 33.2, 36.6, 34.6, 33.2	11.2, 10.3, 9.90, 11.2, 10.3, 9.90	37.8, 27.9, 27.9, 30.8, 27.9, 27.9	28.9, 30.6, 30.2, 28.9, 30.6, 30.3
	CS	35.8, 32.2, 41.0,	11.4, 10.0, 13.1	18.6, 6.80, 19.7	66.4, 84.3, 58.9
[**2**(**1B**)$_6$]	PP	33.1, 37.3, 34.5, 33.8, 36.7, 35.2	9.82, 11.3, 10.3, 10.0, 11.2, 10.4	11.0, 35.3, 11.5, 33.4, 12.9, 34.0	26.4, 15.7, 26.9, 16.8, 26.9, 15.6
	QP	33.8, 33.3, 35.3, 33.8, 37.3, 35.3	10.0, 11.3, 10.4, 10.0, 11.3, 10.4	33.4, 35.3, 34.0, 33.4, 35.3, 34.0	15.6, 15.6, 16.7, 15.7, 15.5, 16.7
	CS	35.0, 35.6, 35.3	10.9, 11.3, 11.4	16.7, 16.4, 18.9	1.52, 1.35, 7.63, 1.52, 1.35, 7.63
[**2**(**1C**)$_6$]	PP	37.0, 34.9, 33.4, 37.0, 34.8, 33.5	11.2, 10.3, 9.90, 11.2, 10.3, 9.90	22.4, 28.9, 20.3, 30.5, 20.8, 28.4	12.0, 1.26, 10.8, 1.21, 11.8, 2.46
	QP	37.0, 34.8, 33.4, 37.0, 34.8, 33.5	11.2, 10.3, 9.90, 11.2, 10.3, 9.90	20.9, 19.6, 19.0, 20.9, 19.6, 19.0	0.29, 0.99, 2.69, 0.29, 1.02, 2.64
	CS	34.7, 37.3, 35.5	10.8, 10.9, 11.3	9.50, 39.7, 14.7	27.0, 16.1, 16.3
[**2**(**1D**)$_6$]	PP	37.0, 34.9, 33.4, 37.0, 34.8, 33.5	11.2, 10.3, 9.90, 11.2, 10.3, 9.90	22.4, 28.8, 20.3, 30.5, 20.8, 28.4	11.9, 1.31, 10.8, 1.28, 11.8, 2.46
	RQ	37.0, 34.8, 33.4, 37.0, 34.8, 33.4	11.2, 10.3, 9.90, 11.2, 10.3, 9.90	20.9, 19.6, 19.0, 20.9, 19.6, 19.0	0.35, 0.90, 2.64, 0.38, 0.97, 2.61
	SP	37.0, 34.9, 33.5, 37.0, 34.9, 33.5	11.2, 10.3, 9.90, 11.2, 10.3, 9.90	30.5, 28.8, 28.4, 30.5, 28.8, 28.4	1.28, 1.31, 2.46, 1.28, 1.34, 2.46
	CS (part 1)	31.7, 36.3, 35.7	9.70, 10.7, 10.8	8.80, 11.9, 33.6	21.9, 15.0, 1.51
	CS (part 2)	34.7, 32.9, 30.5	10.6, 10.1, 9.80	9.60, 13.7, 31.8	28.0, 22.5, 24.9

The SAXS spectrum and its corresponding electron pair-pair distribution function P(r), were first determined theoretically for a selection of the static structures for each of the four [7]rotaxanes. The models selected are visually distinct conformers (Figures S6 – S9), constructed from the low energy conformations of each thread such that the final model was not too tightly packed to have a reasonable expectation of being formed. Whilst each of the thread conformations are likely to be found in solution, it should be noted that once coordinated to the $\{Ni_{12}\}$ core, and the anionic rings are threaded, the resulting structure has relatively little freedom to interconvert between conformers without dissociating to do so.

The results demonstrate that the entire structure appears to be stable in solution. We compared the experimental SAXS and pair distribution functions $P(r)$ (PDF) (Figures S12 - 15) with the predicted SAXS for an ensemble of structures obtained during the MD simulation and then examined how SAXS and PDF would vary through the rotated conformers. The best agreement with experimental data is found for the MD ensemble average. The other calculations are not consistent with the experimental observations as a static representation of each structure's electron distribution is significantly higher at particular distances signifying the extra peaks around the central averaged one seen experimentally and in MD.

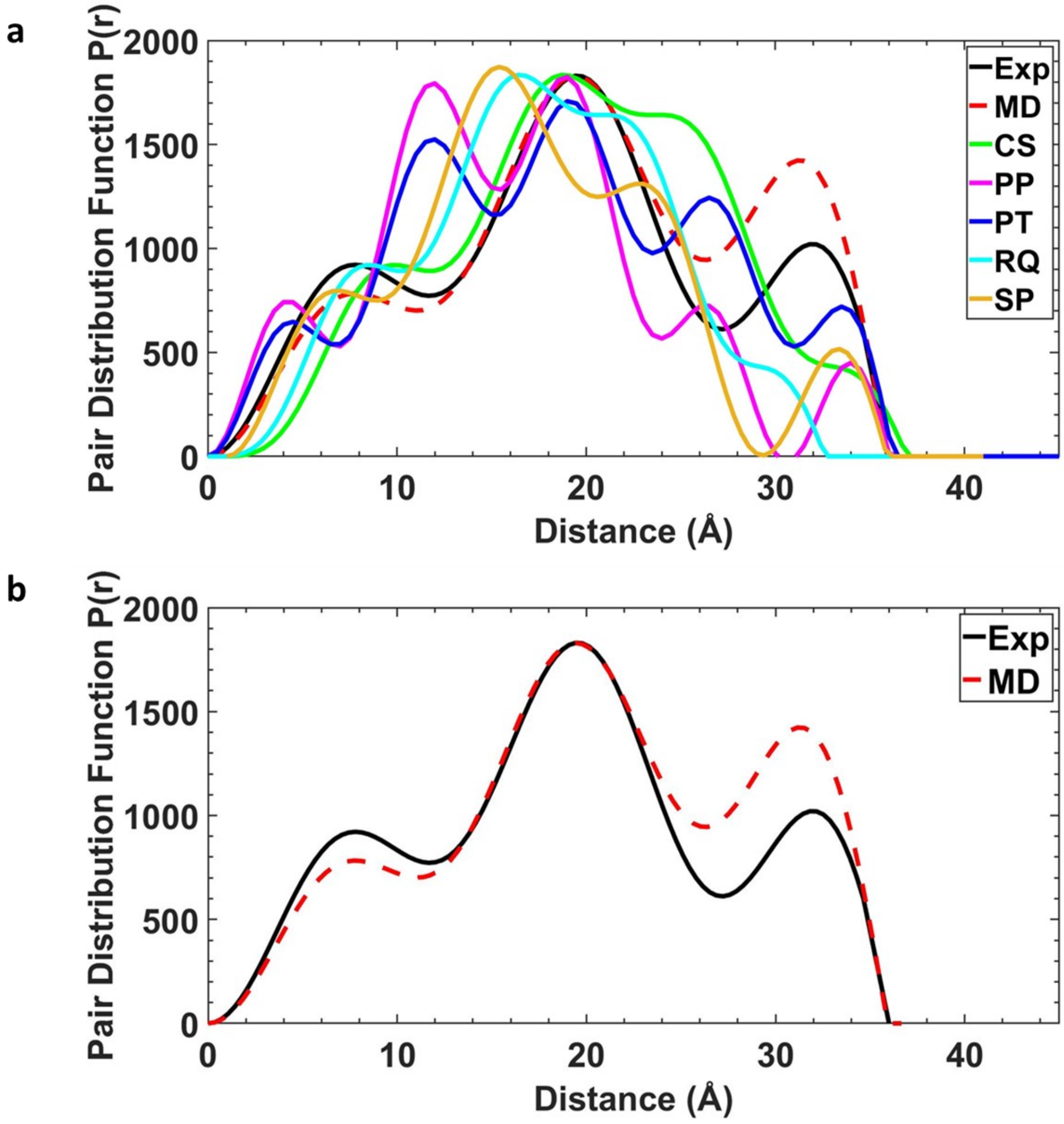

Figure S12. a, Pair pair distribution for various conformers of [**2**(**1A**)$_6$] (pink, blue, cyan, orange) alongside the crystal structure (bright green), experimental (black) and MD (red). b, MD versus experimental for clarity. Various conformers selected illustrate the most obvious differences that can be seen when visually inspected.

a

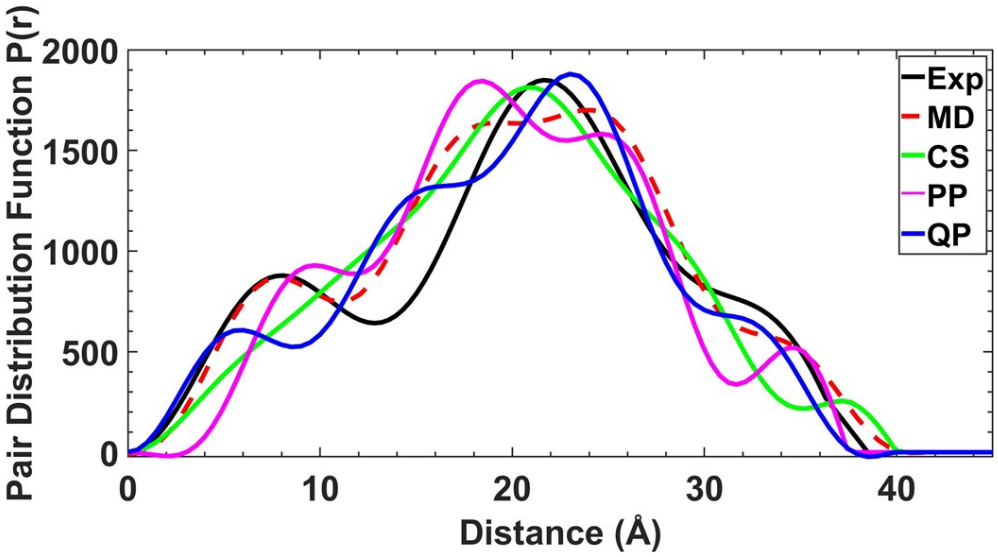

b

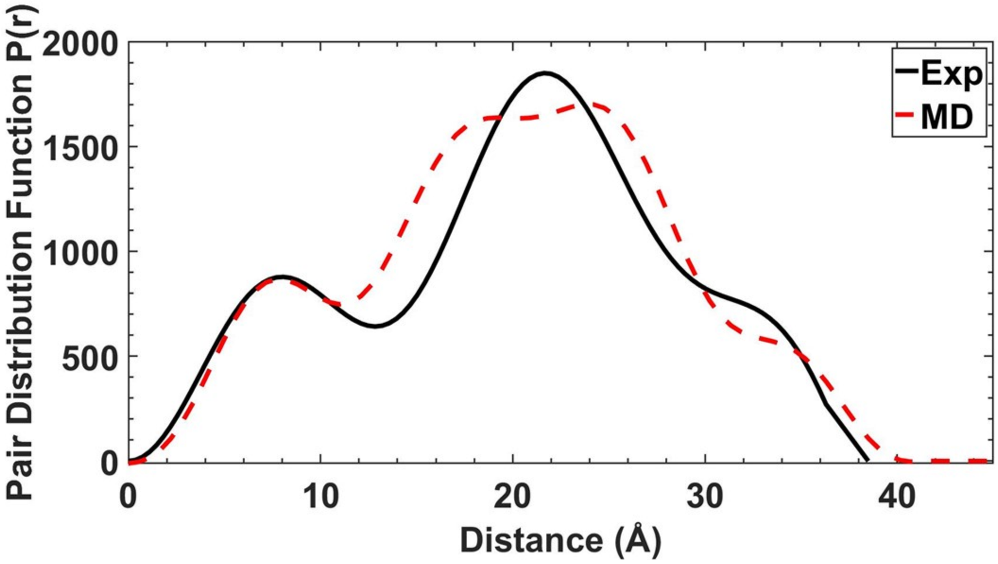

Figure S13. a, Pair pair distribution for various conformers of [**2**(**1B**)$_6$] (pink, blue) alongside the crystal structure (bright green), experimental (black) and MD (red). b, MD versus experimental for clarity. Various conformers selected illustrate the most obvious differences that can be seen when visually inspected.

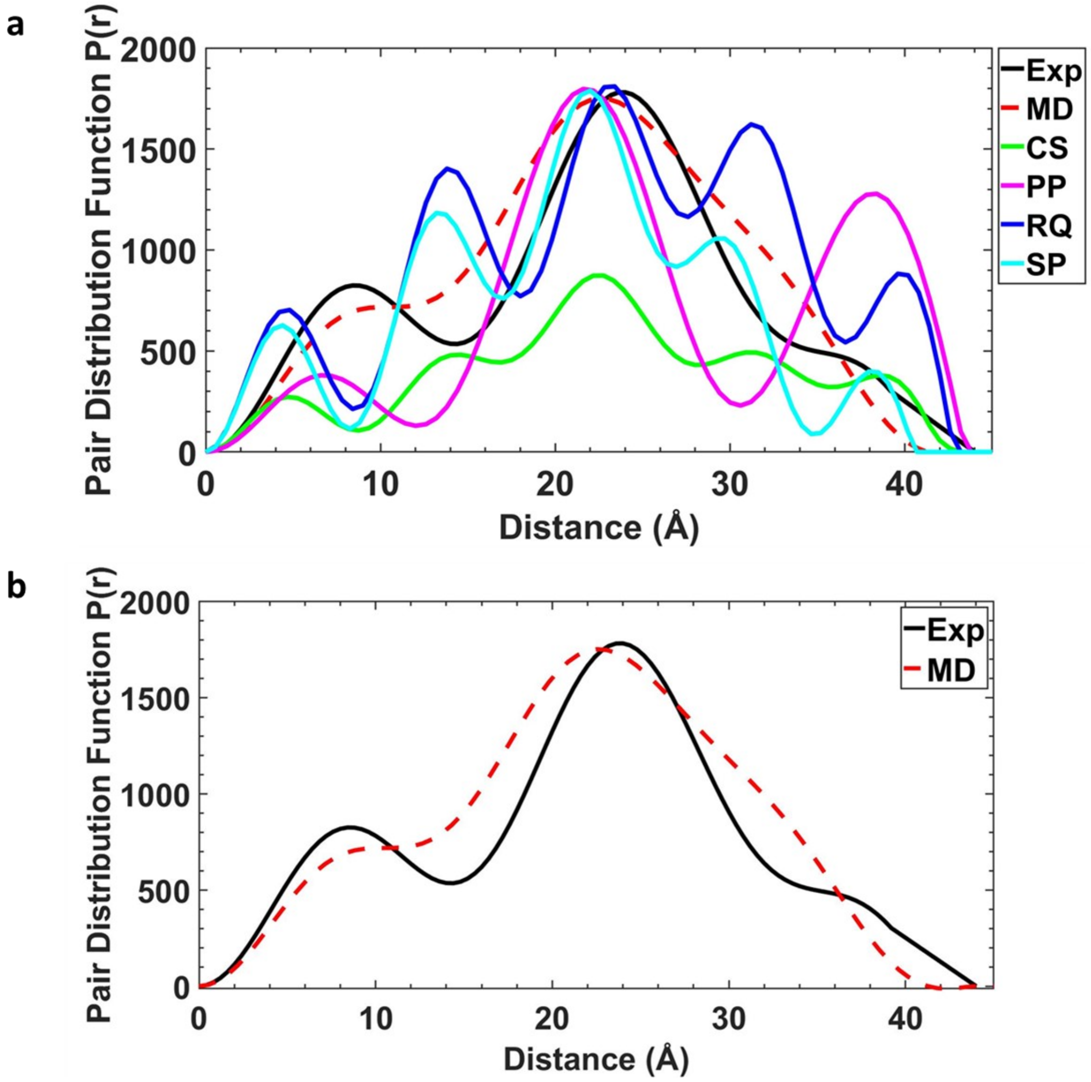

Figure S14. a, Pair pair distribution for various conformers of [**2**(**1C**)$_6$] (pink, blue, cyan) alongside the crystal structure (bright green), experimental (black) and MD (red). b, MD versus experimental for clarity. Various conformers selected illustrate the most obvious differences that can be seen when visually inspected.

a

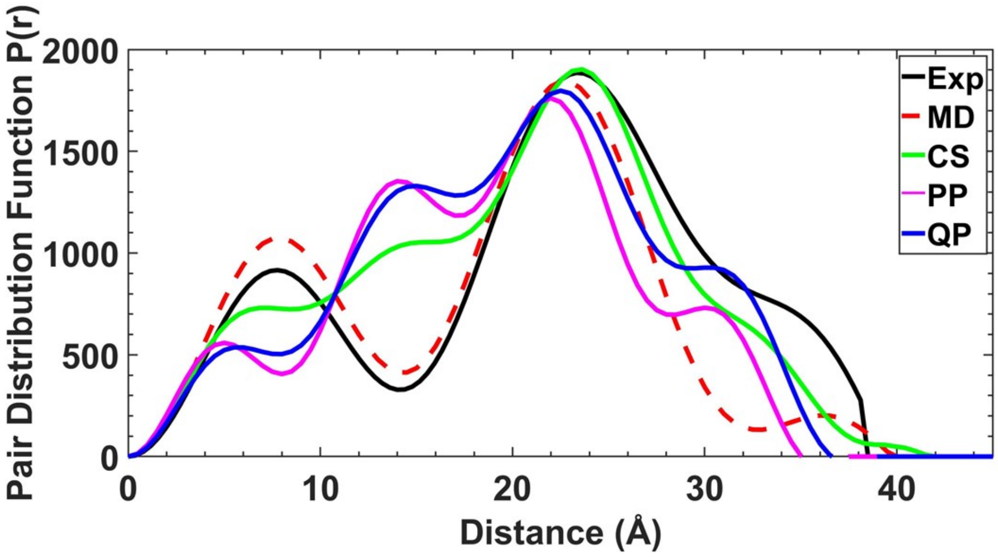

b

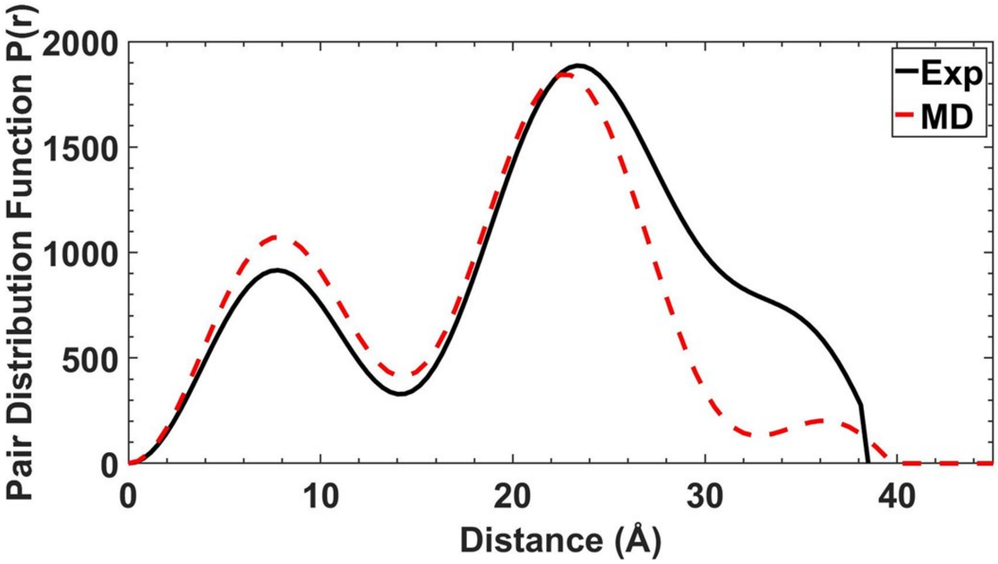

Figure S15. a, Pair pair distribution for various conformers of [**2**(**1D**)$_6$] (pink, blue) alongside the crystal structure (bright green), experimental (black) and MD (red). b, MD versus experimental for clarity. Various conformers selected illustrate the most obvious differences that can be seen when visually inspected.

EPR Spectroscopy

Q-band data were recorded on a Bruker E500 spectrometer equipped with an ER 5106 QT (Q-band, *ca*. 34 GHz) resonator.

Continuous wave (CW) EPR spectra of [**2**(**1A**)$_6$], [**2**(**1B**)$_6$], [**2**(**1C**)$_6$] and [**2**(**1D**)$_6$] were measured at 5 K, both in the solid state and as a frozen solution (1 mM in dry 1:1 CH_2Cl_2:toluene). The spectra are dominated by the S = ½ ground states of the {Cr_7Ni} rings (signal at ca. 13,750 G in Figure S16). The *g*-tensor in all cases g_{xyz} = 1.79, 1.79, 1.75. There is a broad feature at *ca.* 10000 G, which may be due to resonances from **2**. The narrowness of the EPR signal indicates no significant magnetic interactions between **2** and **1X** in any compound. Figure S17 shows a broad feature observed when measuring the powder CW EPR spectrum of [**2**(THF)$_6$] at 5 K.

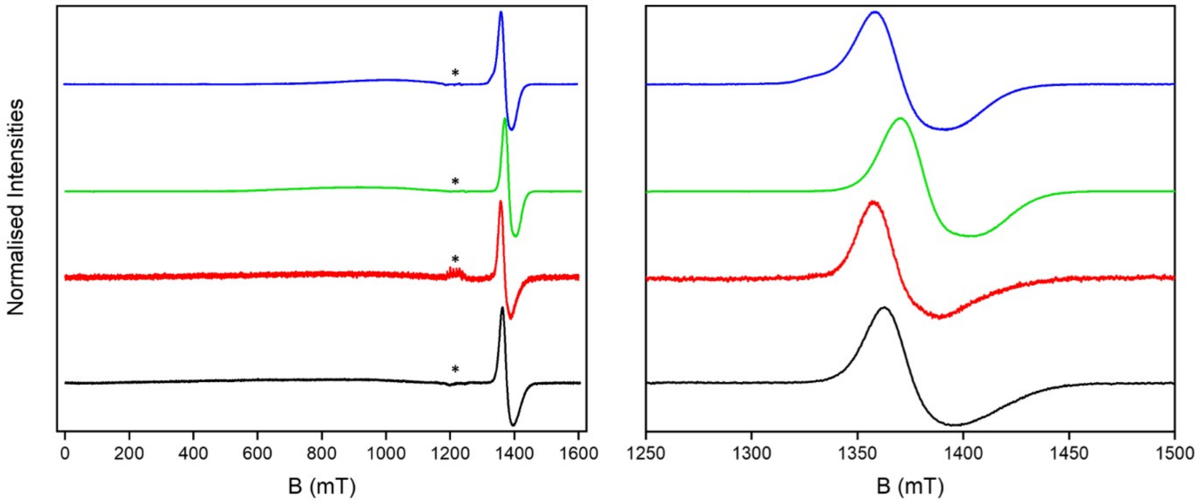

Figure S16. Solution CW Q-band EPR for [**2**(**1A**)$_6$] (black), [**2**(**1B**)$_6$] (red), [**2**(**1C**)$_6$] (green) and [**2**(**1D**)$_6$] (blue).* indicates a Mn^{II} impurity in the EPR tube (quartz).

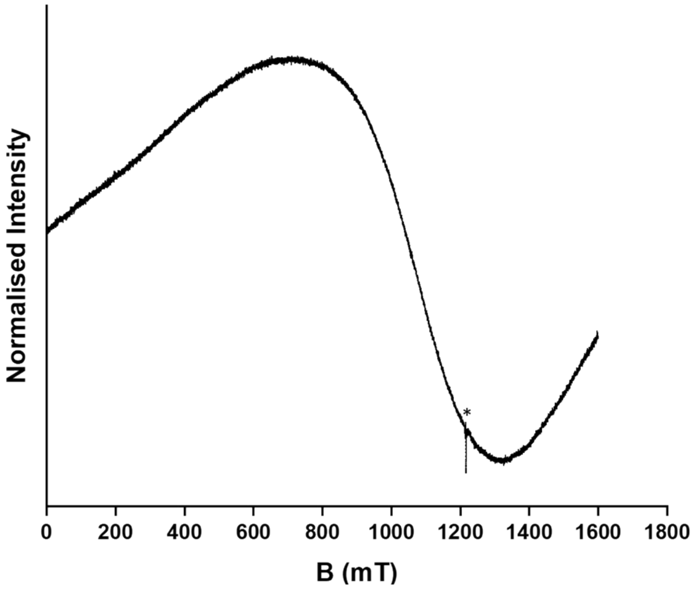

Figure S17. Powder CW Q-band EPR for [**2**(THF)$_6$]. * indicates a Mn^{II} impurity in the EPR tube (quartz).

Pulsed EPR (Q-band, 3 K) was used to measure the phase memory times (T_m) for [**2**(**1A**)$_6$], [**2**(**1B**)$_6$], [**2**(**1C**)$_6$] and [**2**(**1D**)$_6$] (0.1 mM toluene solutions) using standard Hahn echo decay measurements [π/2–τ–π–τ–echo] (Figure S19 – 22). Spin–lattice relaxation (T_1) measurements were carried out by inversion recovery [π–T–π/2–τ–π–τ–echo] (Figure S23 – 26). Measurements were made at magnetic fields (B_0) corresponding to the maximum, cross-over point and minimum of the first derivatives of the echo detected field sweep spectra of the {Cr_7Ni} resonances. The results are given in Table S4. The values observed show no significant variation between compounds nor from isolated {Cr_7Ni} rings.

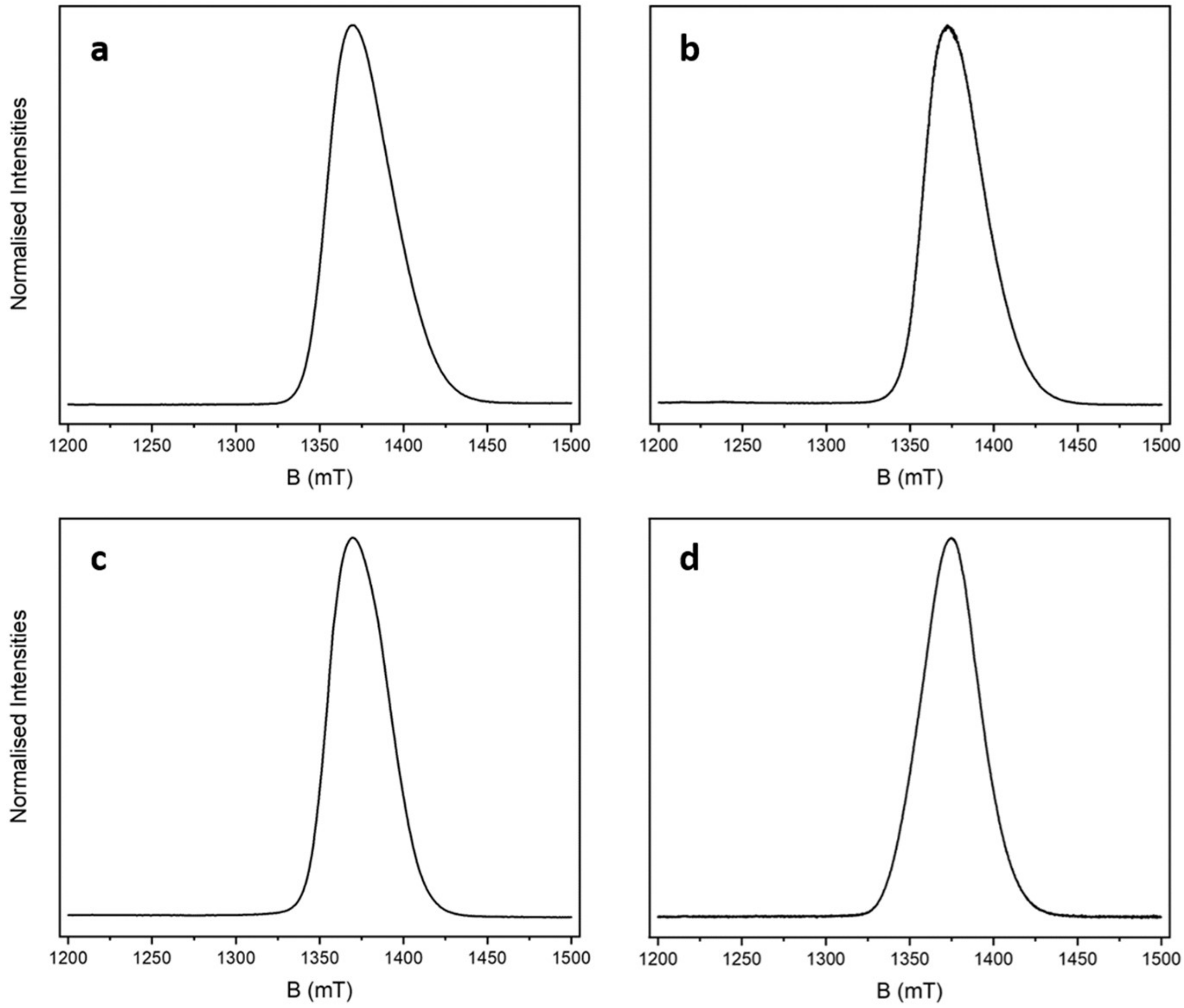

Figure S18. Q-band echo-detected field swept EPR spectrum of, [**2**(**1A**)$_6$]; b, [**2**(**1B**)$_6$]; c, [**2**(**1C**)$_6$]; and d, [**2**(**1D**)$_6$]. Spectra are measured 3 K using 0.1 mM (toluene) solutions.

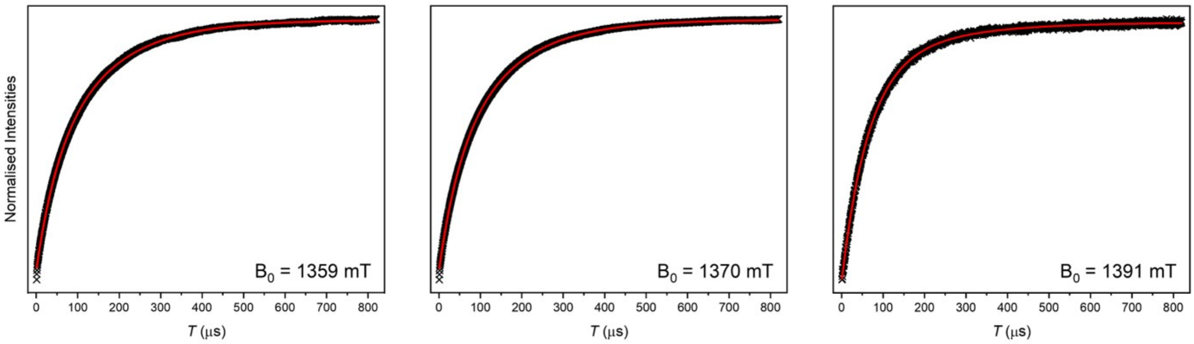

Figure S19. Pulsed EPR (Q-band, 3 K) spin-lattice relaxation time measurements (T_m) of [**2**(**1A**)$_6$] at three field positions.

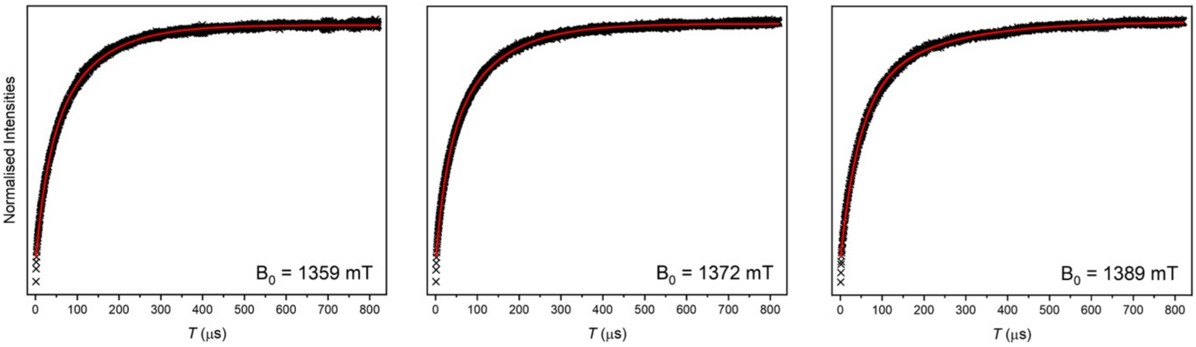

Figure S20. Pulsed EPR (Q-band, 3 K) spin-lattice relaxation time measurements (T_m) of [**2**(**1B**)$_6$] at three field positions.

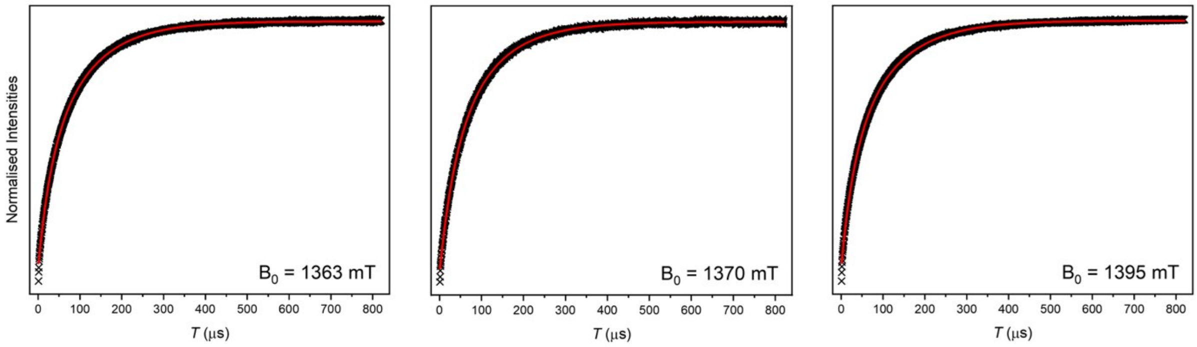

Figure S21. Pulsed EPR (Q-band, 3 K) spin-lattice relaxation time measurements (T_m) of [**2**(**1C**)$_6$] at three field positions.

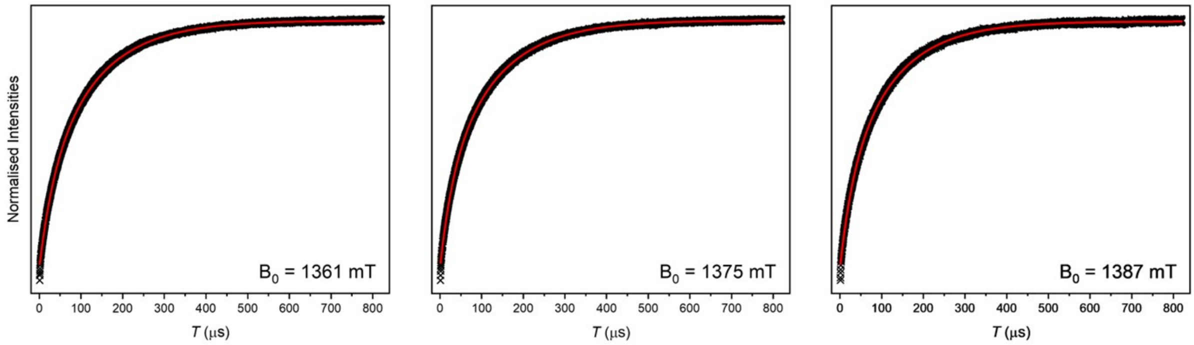

Figure S22. Pulsed EPR (Q-band, 3 K) spin-lattice relaxation time measurements (T_m) of [**2**(**1D**)$_6$] at three field positions.

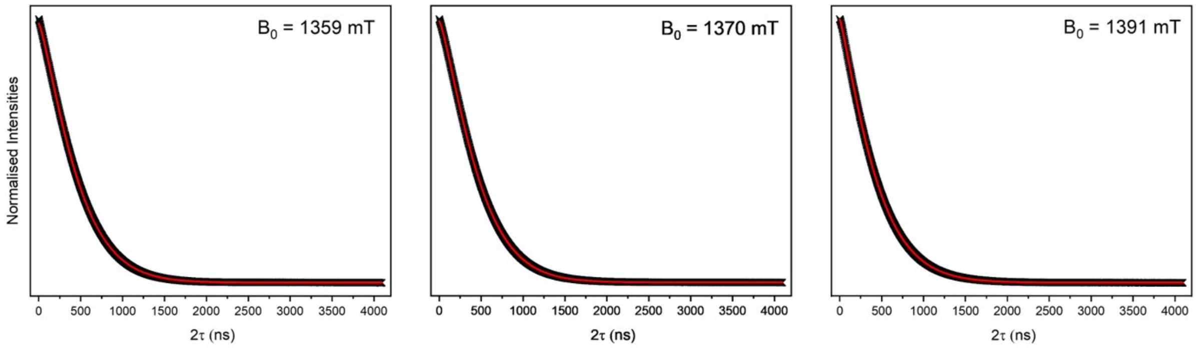

Figure S23. Pulsed EPR (Q-band, 3 K) phase memory time measurements (T_m) of [**2**(**1A**)$_6$] at three field positions.

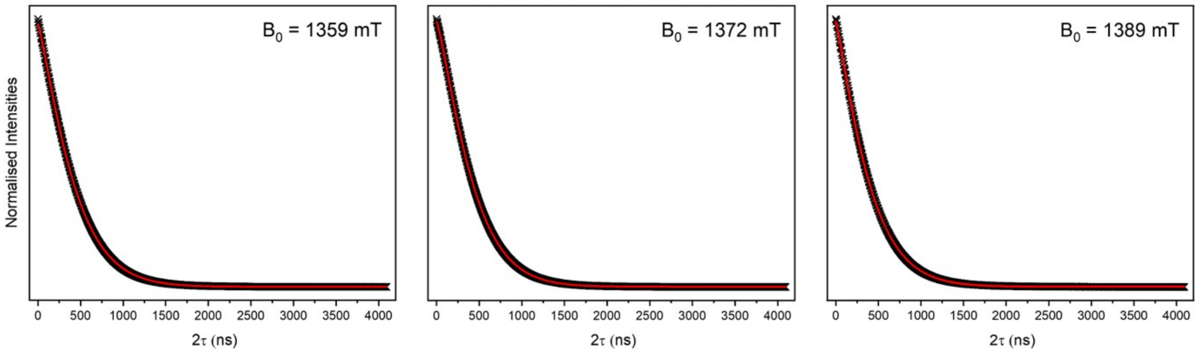

Figure S24. Pulsed EPR (Q-band, 3 K) phase memory time measurements (T_m) of [**2**(**1B**)$_6$] at three field positions.

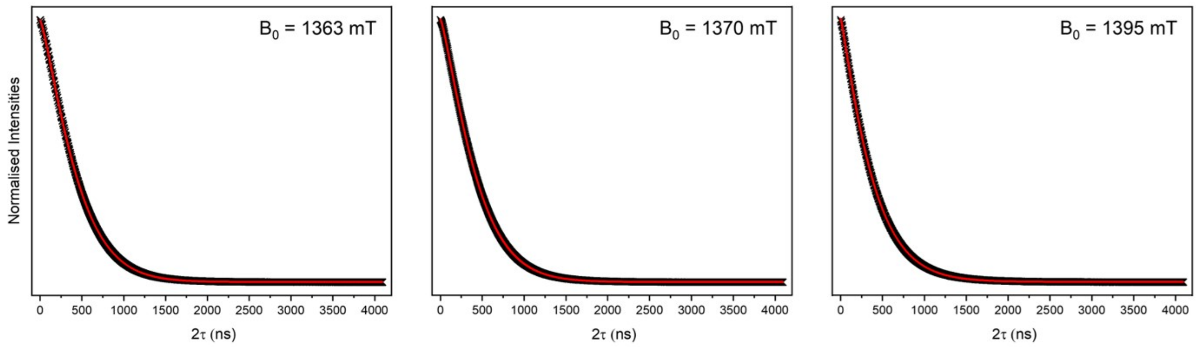

Figure S25. Pulsed EPR (Q-band, 3 K) phase memory time measurements (T_m) of [**2**(**1C**)$_6$] at three field positions.

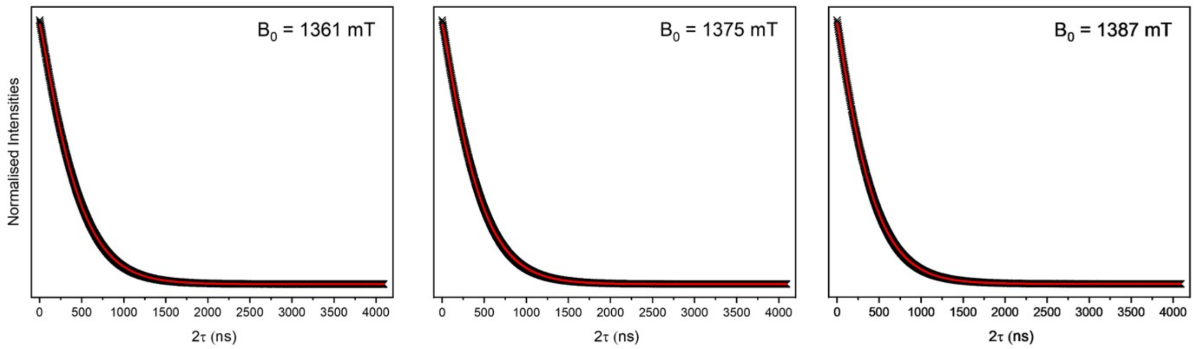

Figure S26. Pulsed EPR (Q-band, 3 K) phase memory time measurements (T_m) of [**2**(**1D**)$_6$] at three field positions.

Table S4. Spin–lattice relaxation (T_1) and phase memory (T_m) times measured for [**2**(**1A**)$_6$] – [**2**(**1D**)$_6$]

B_0, mT	1361		1375		1387	
	T_m, ns	T_1, μs	T_m, ns	T_1, μs	T_m, ns	T_1, μs
[**2**(**1A**)$_6$]	493(0.5)	146(1.0)	500(0.5)	142(1.0)	460(0.5)	191(6.0)
[**2**(**1B**)$_6$]	445(0.5)	109(1.0)	446(0.5)	116(1.0)	415(0.5)	181(3.0)
[**2**(**1C**)$_6$]	428(0.5)	102(1.0)	457(0.5)	106(1.0)	400(0.5)	102(1.0)
[**2**(**1D**)$_6$]	469(0.5)	124(0.5)	414(0.5)	123(0.5)	392(0.5)	116(0.5)

References

1 CrysAlisPRO, Oxford Diffraction /Agilent Technologies UK Ltd, Yarnton, England.

2 G. M. Sheldrick, SHELXL-20xx, University of Göttingen and Bruker AXS GmbH, Karlsruhe (Germany) 2012–2014;

3 O. V. Dolomanov, L. J. Bourhis, R. J. Gildea, J. A. K. Howard and H. Puschmann, *J. Appl. Cryst.*, 2009, **42**, 339-341.

4 Persistence of Vision Raytracer (Version 3.6) Pty. Ltd., Williamstown, Victoria, Australia.

5 J. Ilavsky and P. R. Jemian, *J. Appl. Crystallogr.*, 2009, **42**, 347–353.

6 H. J. C. Berendsen, D. van der Spoel and R. van Drunen, *Comput. Phys. Commun.*, 1995, **91**, 43–56.

7 E. Lindahl, B. Hess and D. van der Spoel, *J. Mol. Model.*, 2001, **7**, 306–317.

8 W. D. Cornell, P. Cieplak, C. I. Bayly, I. R. Gould, K. M. Merz, D. M. Ferguson, D. C. Spellmeyer, T. Fox, J. W. Caldwell and P. A. Kollman, *J. Am. Chem. Soc.*, 1995, **117**, 5179–5197.

9 S. Nosé, *Mol. Phys.*, 1984, **52**, 255–268.

10 M. Parrinello and A. Rahman, *J. Appl. Phys.*, 1981, **52**, 7182–7190.

11 U. Essmann, L. Perera, M. L. Berkowitz, T. Darden, H. Lee and L. G. Pedersen, *J. Chem. Phys.*, 1995, **103**, 8577–8593.

12 D. T. Cromer and J. B. Mann, *Acta Crystallogr. Sect. A*, 1968, **24**, 321–324.
